# An improved blind watermarking scheme for color image copyright protection using Hahn moments

**DOI:** 10.1038/s41598-026-42088-9

**Published:** 2026-04-21

**Authors:** Nada I. Elbatawy, Abdelrahman A. Karawia, Mostafa M. El-Gayar, Yasser M. Fouda

**Affiliations:** 1 Faculty of Science, Mansoura University, Mansoura, 35516 Egypt; 2 Faculty of Computers and Information, Mansoura University, Mansoura, 33516 Egypt; 3 College of Engineering and Information Technology, Onaizah Colleges, Onaizah, Saudi Arabia; 4 Department of Computer Science, Arab East Colleges, Riyadh, 11583 Saudi Arabia

**Keywords:** Blind watermarking, Color image protection, Transformed domain, Hahn moments, Engineering, Mathematics and computing

## Abstract

Ensuring copyright protection against illegal attacks and image processing operations while maintaining blind detection capabilities presents a significant challenge in color image watermarking. To address this issue, in this article, we propose a blind watermarking approach that integrates the Arnold transformation with watermark embedding in the transformed domain. Modified forms of Hahn discrete moments are introduced to effectively extract the key image features within this domain. The watermark is encrypted before being embedded into the magnitudes of the Hahn moments for each block using dither modulation, ensuring robustness against different types of attacks. Additionally, a reconstruction algorithm for color images based on Hahn moments is developed and employed during the watermark extraction phase. Experimental results confirm that the proposed scheme achieves high efficiency in terms of both imperceptibility and robustness. Extensive evaluations demonstrate its superior performance, with its PSNR and SSIM values reaching 64.693 dB and 0.9998, respectively. In the no-attack scenario, a perfect-quality watermark is extracted with BER = 0 and NCC = 1. Under various attacks, including filtering, noise, geometric, and robustness attacks, the proposed scheme continues to extract high-quality watermarks, achieving an average BER of 0.00001 and NCC of 0.9998, thereby outperforming the existing image watermarking techniques.

## Introduction

Communications among people worldwide have become more convenient and faster because of the rapid advancements achieved in computer networks and multimedia processing technologies. Digitizing multimedia data not only facilitates easy access to multimedia information but also significantly enhances the efficiency and accuracy of expressing information. With the rapid advancement of computer-based multimedia technology, people can easily use digital equipment to produce, manipulate, and store various forms of media information, such as text, documents, images, audio, and video. In addition, the transmission of digital data has become increasingly easier and faster to implement because of the widespread use of high-definition imaging technologies and significant advancements in communications technologies. These technologies enable the storage and sharing of enormous volumes of digital data on open platforms such as LinkedIn and Google Drive. As internet technology has developed rapidly, image data have become widely used across various social networks, including Facebook, Twitter (X), and Flickr^1^. Digital images are the most utilized and shared images on these platforms, and they have been extensively used in many aspects of life and work. Doing so has become more essential in contemporary society in recent years^2,3^. Although several digital media products are extensively transmitted online, a series of problems have appeared, such as illegal copying, copyright infringement, and malicious tampering. Digital media products can be easily copied and spread; therefore, it is difficult to distinguish legal copies from illegal copies. The problem of copyright infringement often occurs with regard to digital media. Therefore, under the increasingly strict security of multimedia big data, researchers worldwide have focused on the problem of copyright protection as a research hotspot^4,5^. As an effective copyright protection method, digital watermarking technology plays a pivotal role in the field of multimedia information security and has widespread application prospects^6,7^.

Moreover, in the era of big data, digital images—especially color images—are widely used in daily life, making information storage and processing operations more convenient. However, digital images can be stored, copied, and shared without distortion or alteration and can also be distributed without authorization, posing a serious threat to the healthy development of the information industry. In addition, digital images can be reprinted randomly on the internet, leading to numerous cases of piracy and infringement of the legitimate rights and interests of copyright owners. Consequently, the issue of copyright protection for digital color images is becoming increasingly serious and represents an urgent problem that must be addressed.

Digital watermarking has become a valuable technology and an effective method for protecting the copyright and content authentication of digital images, as it is capable of preventing the unauthorized copying, alteration, and distribution of digital content^8^. Digital watermarking is a security technology that hides information by embedding and extracting data. The fundamental concept of digital watermarking involves embedding an ownership mark as a watermark within the original digital product, which is subsequently transmitted in a secure manner. At the receiving end, the embedded ownership mark is extracted and utilized for various purposes, such as content authentication, digital signatures, and secret communication. Despite the significant attention given to digital watermarking technology and the rapid advancements achieved therein, there is still no clear and uniform theoretical definition of a digital watermark. Fan et al.^9^ defined a watermark as follows: " A digital watermark is embedded information inserted into digital media that is not recognized by the user but is robust to compression, processing, or other forms of manipulation. It serves to verify ownership, ensure content integrity, and track data sources and distribution routes, thereby improving data security and traceability”.

Watermarking techniques can be broadly categorized on the basis of four criteria: perceptibility (visible or invisible)^10^, the domain of embedding (the spatial, frequency, or transform domain), robustness (robust^11^, semi-fragile^12^, or fragile^13^, and the method of data extraction (non-blind^14^, blind^15^, or semi-blind^16^. Visible watermarking embeds a watermark, such as a logo or text, directly onto the target image in a manner that is perceptible to the human eye and is typically used for copyright protection and branding. In contrast, invisible watermarking hides the watermark within the image data, ensuring that it remains imperceptible under normal viewing conditions while still being detectable through specialized extraction techniques. On the basis of the embedding domain, watermarking can be classified into spatial-domain watermarking, where modifications are applied directly to pixel values, and transform-domain watermarking, which embeds the watermark into the frequency components of the target image using transforms such as the discrete cosine transform (DCT), the discrete wavelet transform (DWT), or Hahn moments, thereby offering improved robustness. Additionally, watermarking techniques are categorized on the basis of their resistance to attacks: robust watermarking ensures that the watermark resists image processing operations such as compression, filtering, and geometric distortions, whereas fragile watermarking is designed to detect any modifications, making it suitable for authentication and tamper detection purposes. Semi-fragile watermarking balances robustness and sensitivity by allowing a certain level of acceptable modifications while still detecting unauthorized alterations.

One of the most significant classifications strategies is based on the extraction process, which categorizes watermarking algorithms into blind, non-blind, and semi-blind methods. In blind watermarking, the embedded data are extracted without requiring the original host signal, making it highly practical for applications such as broadcast monitoring and copyright protection. Conversely, non-blind watermarking relies on the availability of the original host signal or watermark for extraction purposes, offering higher robustness and accuracy, and is often used in forensic and authentication tasks. Semi-blind watermarking strikes a balance between these two approaches by utilizing partial information about the original signal or watermark, making it suitable for scenarios in which limited side information is available. Understanding these categories is crucial for selecting an appropriate watermarking technique that is tailored to specific the application requirements and constraints of interest.

Roy et al.^14^ introduced a non-blind color image watermarking method for copyright protection that focuses on perceptual transparency and robustness. Their method uses the luminance–blue difference–red difference (YCbCr) color space, combining the DWT and singular value decomposition (SVD) within a human visual system (HVS) based adaptive additive embedding scheme (AES). By introducing a modified strength parameter (α_mean), the method achieves improved perceptual quality and resistance to attacks. Liu et al.^15^ presented a blind dual watermarking mechanism for color images that combines robust watermarking for copyright protection with fragile watermarking for authentication. A robust watermark is embedded in the YCbCr color space using the DWT, while a fragile watermark is inserted into the RGB components via an improved least-significant-bit (LSB) technique. This dual approach enables blind extraction, ensures both protection and tamper detection, and effectively identifies altered regions while maintaining robustness against various attacks. Palani and Loganathan^16^ suggested a semi-blind watermarking technique based on a convolutional attention-based turtle shell matrix with a two-level embedding process: the first level uses the turtle shell data hiding algorithm, and the second level applies DWT-SVD. The aforementioned methods have several limitations. First, they require access to the original (unwatermarked) image for watermark extraction, which limits their practicality in real-world applications such as copyright protection. Furthermore, their reliance on the YCbCr color space may limit their embedding capacity and robustness compared with those of approaches that utilize all color channels or alternative color spaces. Additionally, these methods can be susceptible to certain geometric attacks and often involve high computational complexity levels, particularly when handling color images.

To address these limitations, this paper introduces the following main contributions:

- A novel enhancement of Hahn moments is proposed for blind color image watermarking, particularly for color medical images, in which modified moment formulations and an optimized coefficient selection process improve the robustness and imperceptibility of the moments without increasing their computational complexity.
- A redesigned embedding–extraction framework that exhibits enhanced stability against common attacks in the health care domain, such as compression, filtering, and geometric distortions, through improved moment-domain coefficient manipulation, is developed.

## Related works

In recent years, blind watermarking techniques for color image copyright protection have garnered significant attention because of their ability to embed and extract watermarks without requiring access to the original image. Numerous approaches have been proposed, leveraging various mathematical transforms and feature extraction methods to enhance their robustness, imperceptibility, and security. Many existing schemes face challenges in terms of balancing robustness against attacks with maintaining high visual quality for the watermarked image. We build upon these advancements in this paper by proposing an improved blind watermarking scheme based on Hahn moments, which offers superior computational efficiency and robustness, particularly for color images. The following section reviews the key contributions in the field and highlights the gaps that are addressed by the proposed approach.

Spatial-domain watermarking algorithms^17^ directly manipulate the pixel values ​​of the carrier image, which has a greater impact on the visual quality of the carrier image but involves relatively low computational complexity. These methods are relatively simple but sensitive to various attacks, such as histogram equalization, sharpening, and scaling, since they are directly correlated with pixel data. Abraham and Paul^17^ suggested dividing a color image into its three channels—red, green, and blue—and embedding a watermark into each channel separately. The watermark is embedded by modifying the pixel values of the image according to a predefined algorithm that considers the characteristics of the human visual system.

Frequency-domain watermarking algorithms^18–24^ operate on the transformed coefficients of the carrier image rather than manipulating direct pixel values using techniques. A watermark is embedded in the frequency domain by modifying the coefficients of the transformed image, enabling a more effective watermarks embedding approach. Compared with spatial-domain watermarking algorithms, frequency-domain watermarking algorithms can achieve better imperceptibility and robustness against common attacks, such as noise and compression, making them more secure. Cedillo-Hernandez et al.^18^ suggested a robust discrete Fourier transform (DFT)-based watermarking algorithm using spread spectrum and particle swarm optimization (PSO). The method automatically optimizes the key parameters, including frequency bands, coefficients, and the watermark strength using visual information fidelity (VIF) and bit correctness rate (BCR) criteria. This approach exhibits enhanced resistance to signal processing and geometric attacks while maintaining high visual quality. Yuan et al.^19^ introduced a blind color image watermarking method using the 2D-DCT, in which a watermark is embedded and extracted by modifying the relationships between selected middle-frequency coefficients, ensuring invisibility and robustness. Unlike most techniques that use grayscale or binary watermarks, this approach uses color images for both the host and the watermark. Abdulrahman et al.^20^ presented a robust color image watermarking method based on the DCT and DWT, in which the DCT component of each watermark part is embedded into four DWT bands of the color components of the host image. The experimental results demonstrated that their proposed method is robust against linear and non-linear attacks and that the transparency of the watermarked images is protected. Hu et al.^21^ introduced an improved blind color image watermarking algorithm based on SVD with mixed modulation. Their method addresses the common limitations of the traditional SVD-based schemes and significantly improves upon both their imperceptibility and robustness, achieving superior performance at a payload capacity of 1/16 bits per pixel. Su et al.^22^ analyzed a blind color image watermarking scheme based on the upper Hessenberg matrix derived from the Hessenberg transform. The experimental results demonstrated the effectiveness of the technique in terms of its watermark invisibility, robustness against common image processing attacks, and computational efficiency. Su et al.^23^ suggested a blind color image watermarking algorithm that combines an affine transformation for watermark encryption with a Schur decomposition scheme for embedding. The eigenvalues of the decomposed matrix are quantized across different color channels to ensure their invisibility, security, and robustness. The affine transformation provides a large key space, whereas Schur decomposition reduces the incurred computational complexity. Su et al.^24^ presented a color image watermarking method using ternary coding and QR decomposition. The color watermark is encoded into ternary information and embedded into the orthogonal matrix Q of QR-decomposed 3 × 3 host image blocks. This method ensures imperceptibility, high embedding capacity, and real-time performance. Table 1 summarizes the most recent watermarking schemes that have been reported in the literature and compares them in terms of their objectives, methodologies, novelty, and pros and cons. This overview highlights the diversity of the available approaches and helps identify the research gaps that motivate the development of the proposed method.

**Table 1:** Overview of different color image watermarking techniques.

Refs.	Objective	Methodology	Novelty	Pros and cons
Fouda ^36^	To reconstruct multi-channel images and use them to design a semi-blind watermarking scheme	Discrete moment-based approaches, Krawtchouk polynomials	Design of a reconstruction algorithm for multi-channel images in the transform domain based on discrete Krawtchouk polynomials, which is further utilized for efficiently embedding watermarks and extracting them from diabetic retinopathy images	Pros: Accurately reconstructs multi-channel images in the transform domain with a very low MSE (1 × 10⁻⁶); the watermarking scheme is robust against median filtering attacks. Cons: High computational cost, with the total embedding and extraction time reaching 6.19 s, due to the use of Krawtchouk moments.
Singh et al^37^.	To provide video watermarking capabilities based on a meta-heuristic algorithm for securing images	Variant of the gravitational search algorithm that embeds a watermark logo into maximum entropy blocks with sizes of 8 × 8	Introduction of the modified gravitational search algorithm (MGSA) to balance imperceptibility and robustness and embedding in maximum entropy blocks of motion frames for enhanced security	Pros: High resistance to different types of attacks; superior balance between imperceptibility and robustness. Cons: High computational overhead due to video frame processing and meta-heuristic optimization tasks.
Ujwala et al^38^.	To develop an efficient watermarking method that enhances the resilience of images against manipulation and tampering	Utilizes the DWT on the blue channel, encrypts the watermark using a Henon map, and embeds it via alpha blending in high-energy blocks	Use of energy-based block selection within the high-frequency sub-band (HH) to ensure an adaptive embedding that optimizes robustness	Pros: Simple to implement, with high security through encryption. Cons: Vulnerability to some geometric attacks; limited to a single-color channel.
Singh et al^39^.	To secure electronic patient records (EPR) by ensuring integrity and authenticity while mitigating the false-positive problem	Utilizes a hybrid integer wavelet transform (IWT) -SVD domain where a logo is encrypted via a diffused Mandelbrot set-Arnold (DMA) map and embedded into the principal component (PC) using non-dominated sorting genetic algorithm II (NSGA-II) for multi-objective optimization	Use of NSGA-II to find multiple optimal embedding factors (MOEFs) and the DMA encryption scheme to eliminate false-positive issues and enhance security without dividing the image into a region of interest (ROI) and a region of noninterest (RONI)	Pros: Eliminates false-positive errors; lossless watermark recovery; enhanced security through DMA encryption. Cons: Complex implementation due to the use of multi-objective optimization (NSGA-II).
Sharma et al. ^40^	To develop a secure and robust adaptive watermarking scheme where both the host and the watermark are color images with the same dimensions	Uses the RDWT and SVD, and both images are scrambled using the Arnold transform, where the artificial bee colony (ABC) algorithm is used to optimize the adaptive multi-scaling factors based on quality metrics of both the host and watermark	Integration of the RDWT-SVD domain watermark embedding with ABC optimization for developing an adaptive strength factor, which dynamically balances imperceptibility and robustness, and the use of a full-dimension color watermark with chaotic scrambling for enhanced security	Pros: Robust against several image processing attacks; high embedding capacity; shift-invariant. Cons: Increased computational complexity due to the RDWT, SVD, and ABC optimization.
Singh et al^41^.	To provide a comprehensive review and systematic analysis of the evolution of watermarking from classic methods to intelligent soft computing techniques	A systematic survey of spatial and transform-domain techniques combined with an analysis of meta-heuristic optimization algorithms for optimizing multiple embedding factors (MEF)	Provides a detailed classification of soft computing integration schemes in watermarking, identifying meta-heuristic algorithms as the primary solutions for the non-deterministic polynomial-time (NP) complete problem of balancing conflicting parameters	Pros: Extensive roadmap for researchers; provides a deep comparative analysis of various optimization algorithms. Cons: Does not propose a new technical algorithm; high-level theoretical focus.
Sharma et al. ^42^	To design a secure and robust color image watermarking scheme that can resist common signal processing and geometric attacks while maintaining high image quality	The method embeds a watermark into a color image using transform-domain techniques combined with nature-inspired optimization algorithms (such as genetic algorithms, particle swarm optimization, or other bioinspired intelligence methods) to optimize their embedding strength and robustness	Integration of nature-inspired intelligence to adaptively optimize watermark embedding parameters for color images, achieving a balance between imperceptibility and robustness	Pros: High robustness against attacks, improved imperceptibility, adaptive optimization, and suitability for copyright protection. Cons: Increased computational complexity, higher execution time than those of the traditional watermarking methods, and dependence on optimization parameter tuning.
Singh et al^43^.	To protect patient data and secure medical images against attacks while improving upon the security of the traditional methods	Employs a 2-level DWT combined with the advanced encryption standard (AES) to encrypt both the target medical image and patient records before embedding	Replacing the traditional Arnold transform with AES encryption to significantly increase the level of security and using a multilevel DWT to ensure higher robustness and imperceptibility.	Pros: Extremely high security (military grade), very robust against noise. Cons: Increased computational overhead due to the AES algorithm.
Kumar et al^44^.	To ensure image authenticity and copyright protection by balancing the trade-off between imperceptibility and robustness	An adaptive hybrid scheme using the DWT, the Walsh Hadamard transform (WHT), and SVD in the YCbCr space. It adaptively calculates embedding factors via visual and edge entropy and secures the watermark with the Arnold transform	Calculation of the adaptive scaling factor using visual entropy and edge entropy to optimize the embedding strength	Pros: High imperceptibility and robust against common signal processing attacks (noise/filtering). Cons: Its computational complexity is high due to the triple transform (DWT-WHT-SVD).
Sharma et al. ^45^	To develop an efficient and robust color image watermarking scheme that ensures high imperceptibility and robustness against common attacks	The host color image is transferred into the DCT domain, where non-negative matrix factorization (NNMF) is applied to decompose the image components. An optimization technique is used to determine the optimal embedding parameters for inserting the watermark into the selected DCT coefficients	Combining the use of NNMF with an optimization-based strategy in the DCT domain for color image watermarking, enhancing the feature representation and adaptive watermark embedding processes	Pros: Good trade-off between robustness and imperceptibility, effective frequency-domain embedding, improved resistance to signal processing attacks. Cons: Higher computational cost due to NNMF and the optimization process, sensitivity to the selected parameters, and increased algorithmic complexity.
Singh et al^46^.	To achieve high embedding capacity and robust recovery while balancing the trade-off between imperceptibility and robustness in color images	Uses redundant discrete wavelet transform (RDWT)-Schur decomposition, which encrypts the watermark with the Arnold transform and optimizes multiple embedding factors using self-adaptive biogeography-based optimization (SBBO)	Hybridization of the RDWT and Schur transform to provide shift invariance and high capacity and the use of SBBO ensures a faster and more precise search for the optimal embedding factors than that provided by standard BBO	Pros: High embedding capacity, shift invariance, and high robustness against various attacks. Cons: Computationally intensive due to the meta-heuristic optimization process.

Moments are pivotal mathematical constructs that are used in image processing tasks to describe various features of images and play a crucial role in the field of watermarking. Orthogonal moment families are classified into two major categories: continuous orthogonal moments (COMs) and discrete orthogonal moments (DOMs). COMs are powerful mathematical image representation and analysis tools in various fields that arise from the continuous representation of an image and are typically defined over a continuous domain. The complexity of COMs requires sophisticated mathematical understanding and computational resources, making them less practical for real-time applications.

DOMs are derived from sampled image data, making them particularly useful in image processing scenarios, as they can efficiently handle noisy or incomplete data while maintaining computational simplicity. They are computed using summations rather than integrals, which simplifies the process and makes them more accessible for various applications. Discrete moments are critical for real-time applications, as they are easier to implement and can provide sufficient accuracy for numerous tasks in image analyses. The discrete nature of these moments enables their direct application in digital image processing cases by converting continuous image functions into a finite set of discrete values. Ernawan and Kabir^25^ introduced a block-based watermarking technique using Tchebichef moments (TMs) for copyright protection purposes. The target image is divided into nonoverlapping blocks, and watermarks are embedded in the low-frequency components to enhance their resistance to distortions. To achieve improved security, the watermarks are encrypted prior to the embedding step. Extraction is performed by computing the TMs of the watermarked image and comparing them with the original moments to detect any alterations or tampering. Venkataramana and Raj^26^ suggested a watermarking scheme that embeds and extracts watermarks using Krawtchouk moments (KMs), where a watermark is embedded into the target image by modifying the KMs of the image. This modification provides robustness against common image processing attacks, such as compression, noise addition, and cropping. Chekira et al.^27^ presented a block-based watermarking method for large-scale medical images using Racah moments and a novel evolutionary algorithm for selecting optimal embedding positions. The watermark includes encrypted biometric and medical data (fingerprints, electronic patient records (EPRs), and the doctor’s face) to ensure confidentiality, integrity, and authentication.

The choice between continuous and discrete orthogonal moments ultimately depends on the specific application of interest and the characteristics of the data being analyzed. While continuous moments may excel at capturing smooth variations, discrete moments provide robustness against noise and computational efficiency. In the context of this study, discrete Hahn moments are utilized for embedding and extracting a watermark image within a color host image. Discrete Hahn moments strike a balance between computational efficiency and robustness, providing high adaptability to various image conditions without compromising the quality of the watermark. This choice is advantageous in scenarios where the watermark must withstand common image manipulations, such as compression or filtering, which could otherwise compromise the integrity of continuous moments. Building upon the analysis of the existing watermarking schemes, an improved blind watermarking method based on Hahn moments that achieves a balance between robustness against attacks and high visual quality for the resulting watermarked image is proposed in this paper.

### **Proposed methodology**

This section presents the proposed techniques for reconstructing and watermarking color images using Hahn moments. Image reconstruction is performed by decomposing and applying the inverse transform to the color channels using Hahn moment coefficients. For watermarking purposes, the watermark is embedded in the Hahn moment domain to ensure both imperceptibility and robustness against common image processing attacks.

### Color image reconstruction

Hahn moments, which are derived from discrete orthogonal Hahn polynomials, are widely used in image analyses because of their high feature representation accuracy. Hahn polynomials ${H}_{n}^{\left(\alpha,\beta\right)}(x,N)$ with orders $(n>N)$ possess parameters $(\alpha,\beta>-1)$ that satisfy the following second-order difference equation:

1 $${\upsigma}\left(x\right)\varDelta\nabla{y}\left(x\right)+\phi\left(x\right)\varDelta{y}\left(x\right)+{\eta}_{n}y\left(x\right)=0,$$

where the Hahn coefficients are given by

2 $$\sigma\left(x\right)=\left(N+\alpha-x\right)x,\phi\left(x\right)=\left(N-1\right)\left(\beta+1\right)-(\alpha+\beta+2)x,\mathrm{a}\mathrm{n}\mathrm{d}{\eta}_{n}=(\alpha+\beta+n+1)n.$$

Then ${H}_{n}^{\left(\alpha,\beta\right)}(x,N)$ can be expressed as shown in Eq. (1):

3 $$\left(N+\alpha-x\right)x\varDelta\nabla{H}_{n}^{\left(\alpha,\beta\right)}(x,N)+(\left(N-1\right)\left(\beta+1\right)-(\alpha+\beta+2\left)x\right)\varDelta{H}_{n}^{\left(\alpha,\beta\right)}(x,N)+(\alpha+\beta+n+1)n{H}_{n}^{\left(\alpha,\beta\right)}(x,N)=0$$

where $x,n=\mathrm{1,2},\dots,N$; $\alpha,\beta>-1$, and $\varDelta{h}\left(x\right)$ and $\nabla{h}\left(x\right)$ refer to the forward and backward difference operators of $h\left(x\right)$, respectively; then,

4 $$\varDelta\nabla{h}\left(x\right)=h\left(x+1\right)-2h\left(x\right)+h\left(x-1\right)$$

The 1-D discrete Hahn orthogonal polynomials of degree $n$ can be expressed in terms of the hypergeometric function as follows:

5 $${H}_{n}^{\left(\alpha,\beta\right)}\left(x,N\right)=\frac{{(-1)}^{n}{(N-n)}_{n}{(\beta+1)}_{n}}{n!}\times3{F}_{2}(-n,-x,n+1+\alpha+\beta;1+\beta,1-N;1)$$

6 $$=\frac{{(-1)}^{n}{(N-n)}_{n}{(\beta+1)}_{n}}{n!}\times\sum_{k=0}^{n}\frac{{(-n)}_{k}{(-x)}_{k}{(n+1+\alpha+\beta)}_{k}}{{(1+\beta)}_{k}{(1-N)}_{k}}\frac{1}{k!}$$

7 $$=\sum_{k=0}^{n}\frac{{\left(-1\right)}^{k}}{k!\left(n-k\right)!}\frac{\left(\beta+n\right)!}{\left(\beta+k\right)!}\frac{\left(\alpha+\beta+n+k\right)!}{\left(\alpha+\beta+n\right)!}{(-x)}_{k.}$$

where ${(-x)}_{k}$ is a Pochhammer function and is given by

8 $${(-x)}_{k}=\prod_{i=1}^{k}(-x+i-1)$$

The Hahn polynomials in Eq. (7) increase rapidly with the degree $n$ and suffer from numerical instability at large values of $n$. To overcome this issue, weighted Hahn polynomials ${\stackrel{\sim}{H}}_{n}^{\left(\alpha,\beta\right)}\left(x,N\right)$ are used in the proposed algorithms as follows:

9 $${\stackrel{\sim}{H}}_{n}^{\left(\alpha,\beta\right)}\left(x,N\right)=\sqrt{\frac{w\left(x\right)}{\rho\left(n\right)}}{H}_{n}^{\left(\alpha,\beta\right)}\left(x,N\right)$$

where the weight function $w\left(x\right)$ is computed using the recurrence relation shown below:

10 $$w\left(x+1\right)=\frac{\left(\beta+1+x\right)\left(N-1-x\right)}{\left(N+\alpha-x-1\right)}w\left(x\right),$$

with the initial condition stating that $w\left(0\right)=\frac{{\Gamma}(\alpha+N){\Gamma}(\beta+1)}{{\Gamma}\left(N\right)}.$.

Similarly, the norm function $\rho\left(n\right)$ is calculated using the following recurrence relation:

11 $$\rho\left(n+1\right)=\frac{\left(\alpha+1+n\right)\left(\beta+1+n\right)\left(\alpha+\beta+N+1+n\right)\left(N-n-1\right)\left(\alpha+\beta+2n+1\right)}{\left(n+1\right)\left(\alpha+\beta+1+n\right)\left(\alpha+\beta+2n+3\right)}\rho\left(n\right),$$

where the initial value is $\rho\left(0\right)=\frac{{\Gamma}(1+\alpha){\Gamma}(1+\beta){\Gamma}(1+\alpha+\beta+n)}{\left(1+\alpha+\beta\right){\Gamma}\left(1+\alpha+\beta\right)\left(N-1\right)!}$.

This new set of discrete weighted Hahn polynomials in Eq. (9) satisfies the following orthogonal condition:

12 $$\sum_{x=1}^{N}{\stackrel{\sim}{H}}_{n}^{\left(\alpha,\beta\right)}\left(x,N\right){\stackrel{\sim}{H}}_{{n}^{{\prime}}}^{\left(\alpha,\beta\right)}\left(x,N\right)={\delta}_{n{n}^{{\prime}}}.$$

Direct computations of weighted Hahn polynomials from Eqs. (7) and (9) are costly; thus, an efficient three-term recurrence relation is used in Algorithm 1. It is expressed as shown below^28^.

13 $${\stackrel{\sim}{H}}_{n}^{\left(\alpha,\beta\right)}\left(x,N\right)=\frac{B\times{D}}{A}{\stackrel{\sim}{H}}_{n-1}^{\left(\alpha,\beta\right)}\left(x,N\right)-\frac{C\times{E}}{A}{\stackrel{\sim}{H}}_{n-2}^{\left(\alpha,\beta\right)}\left(x,N\right).$$

where the coefficients *A*, *B*, *C*, *D*, and *E* are defined as follows:

14 $$A=\frac{\left(\alpha+\beta+n\right)n}{\left(\alpha+\beta+2n\right)\left(\alpha+\beta+2n-1\right)}$$

15 $$B=x-\frac{\left(\alpha+\beta+2N\right)\left({\beta}^{2}-{\alpha}^{2}\right)}{4\left(\alpha+\beta+2n\right)\left(\alpha+\beta+2n-2\right)}-\frac{\alpha-\beta+2N-2}{4}$$

16 $$C=-\frac{(\alpha+n-1)(\beta+n-1)}{(\alpha+\beta+2n-2)}\times\frac{(\alpha+\beta+N+n-1)(N-n+1)}{(\alpha+\beta+2n-1)}$$

17 $$D=\sqrt{\frac{(\alpha+\beta+n)(\alpha+\beta+2n+1)n}{(\alpha+n)(\beta+n)(\alpha+\beta+2n-1)(\alpha+\beta+n+N)(N-n)}}$$

18 $$E=\sqrt{\frac{(\alpha+\beta+2n+1)(\alpha+\beta+n-1)}{(\alpha+\beta+n+N)(\alpha+\beta+2n-3)(\alpha+\beta+n+N-1)}}\times\sqrt{\frac{n(n-1)(\alpha+\beta+n)}{(\alpha+n)(\alpha+n-1)(\beta+n)(\beta+n-1)(N-n)(N-n-1)}},$$

The weighted Hahn polynomials with orders of zero and one can be computed as follows:

19 $${\stackrel{\sim}{H}}_{0}^{\left(\alpha,\beta\right)}\left(x,N\right)=\sqrt{\frac{w\left(x\right)}{\rho\left(0\right)}},{\stackrel{\sim}{H}}_{1}^{\left(\alpha,\beta\right)}\left(x,N\right)=\left[\left(\alpha+\beta+2\right)x-(\beta+1)(N-1)\right]\sqrt{\frac{w\left(x\right)}{\rho\left(0\right)}}$$

The weighted Hahn polynomials in Eq. (9) are orthogonal for one-dimensional (1-D) signals. To apply them to image watermarking tasks (embedding and extraction), they are extended to two-dimensional (2-D) image signals $h(x,y)$ that are defined over the domain $\{\mathrm{1,2},...,N\}\times\{\mathrm{1,2},...,M\}$. The orthogonal basis is given by:

20 $$\left\{{\stackrel{\sim}{H}}_{n}^{\left({\alpha}_{x},{\beta}_{x}\right)}\left(x,N\right).{\stackrel{\sim}{H}}_{m}^{\left({\alpha}_{y},{\beta}_{y}\right)}\left(y,M\right):n=1\dots{N},m=1\dots{M}\right\}$$

This new set of polynomials, as described in Eq. (9), satisfies the orthogonality condition:

21 $$\sum_{x=1}^{N}\sum_{y=1}^{M}{\stackrel{\sim}{H}}_{n}^{\left({\alpha}_{x},{\beta}_{x}\right)}\left(x,N\right){\stackrel{\sim}{H}}_{m}^{\left({\alpha}_{y},{\beta}_{y}\right)}\left(y,M\right).{\stackrel{\sim}{H}}_{{n}^{{\prime}}}^{\left({\alpha}_{x},{\beta}_{x}\right)}\left(x,N\right){\stackrel{\sim}{H}}_{{m}^{{\prime}}}^{\left({\alpha}_{y},{\beta}_{y}\right)}\left(y,M\right)={\delta}_{n{n}^{{\prime}}}{\delta}_{m{m}^{{\prime}}}$$

The image moments ${Q}_{nm}$, which are of order $(n+m)$, represent the projection of the image function $h(x,y)$ onto the weighted Hahn polynomials. These Hahn moments are employed in the embedding procedure to insert the watermark into the transform domain and are computed as follows:

22 $${Q}_{mn}=\sum_{x=1}^{N}\sum_{y=1}^{M}h\left(x,y\right).{\stackrel{\sim}{H}}_{n}^{\left({\alpha}_{x},{\beta}_{x}\right)}\left(x,N\right).{\stackrel{\sim}{H}}_{m}^{\left({\alpha}_{y},{\beta}_{y}\right)}\left(y,M\right).$$

By applying the orthogonality condition derived from Eq. (21) and solving Eq. (22) for$h(x,y)$, the 2-D image $h(x,y)$ can be reconstructed in Algorithm 2 in terms of its Hahn moments as follows:

23 $$\widehat{h}\left(x,y\right)=\sum_{n=1}^{N}\sum_{m=1}^{M}{Q}_{mn}.{\stackrel{\sim}{H}}_{n}^{\left({\alpha}_{x},{\beta}_{x}\right)}\left(x,N\right).{\stackrel{\sim}{H}}_{m}^{\left({\alpha}_{y},{\beta}_{y}\right)}\left(y,M\right).$$

Consider a color image , where are the spatial coordinates and represent the RGB channels. The goal is to reconstruct using weighted Hahn polynomials, which are achieved through the following three algorithms.

**Algorithm 1:**
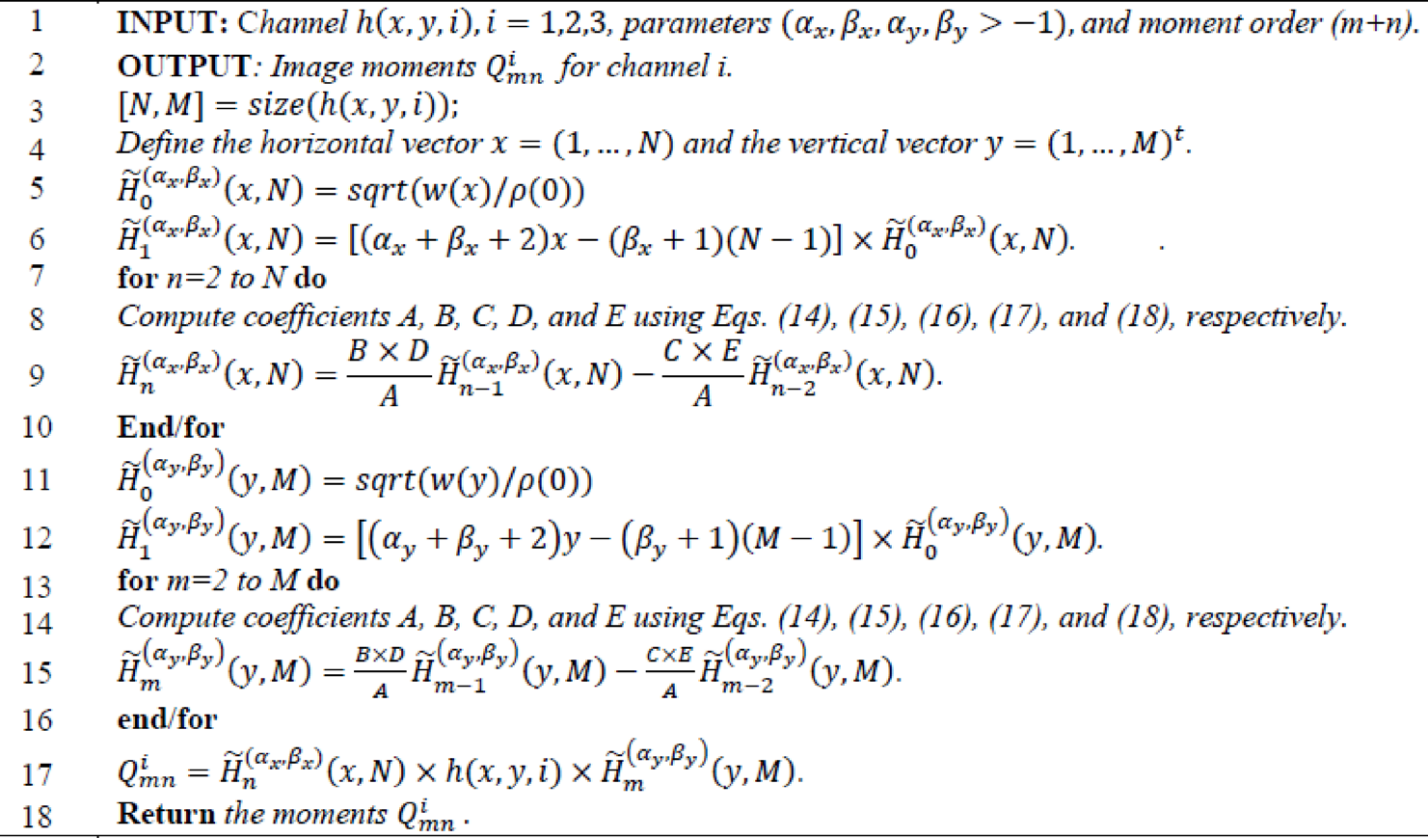
Calculating Hahn moments using their recurrence relation.

**Algorithm 2:**
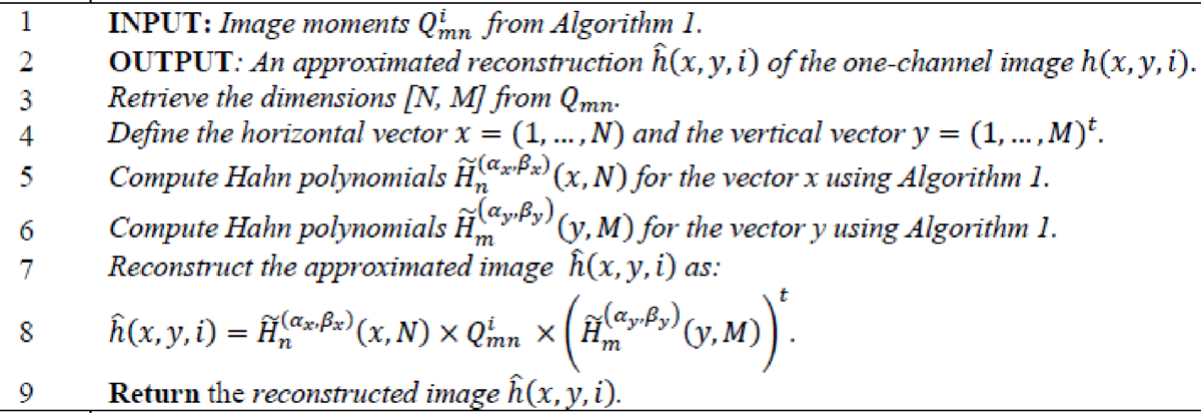
Inverse Hahn moments for a one-channel image.

**Algorithm 3:**
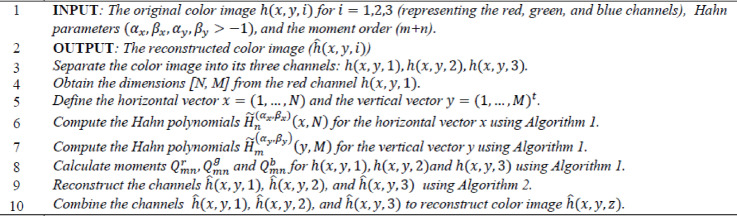
Color image reconstruction using Hahn polynomials.

**Fig. 1:**
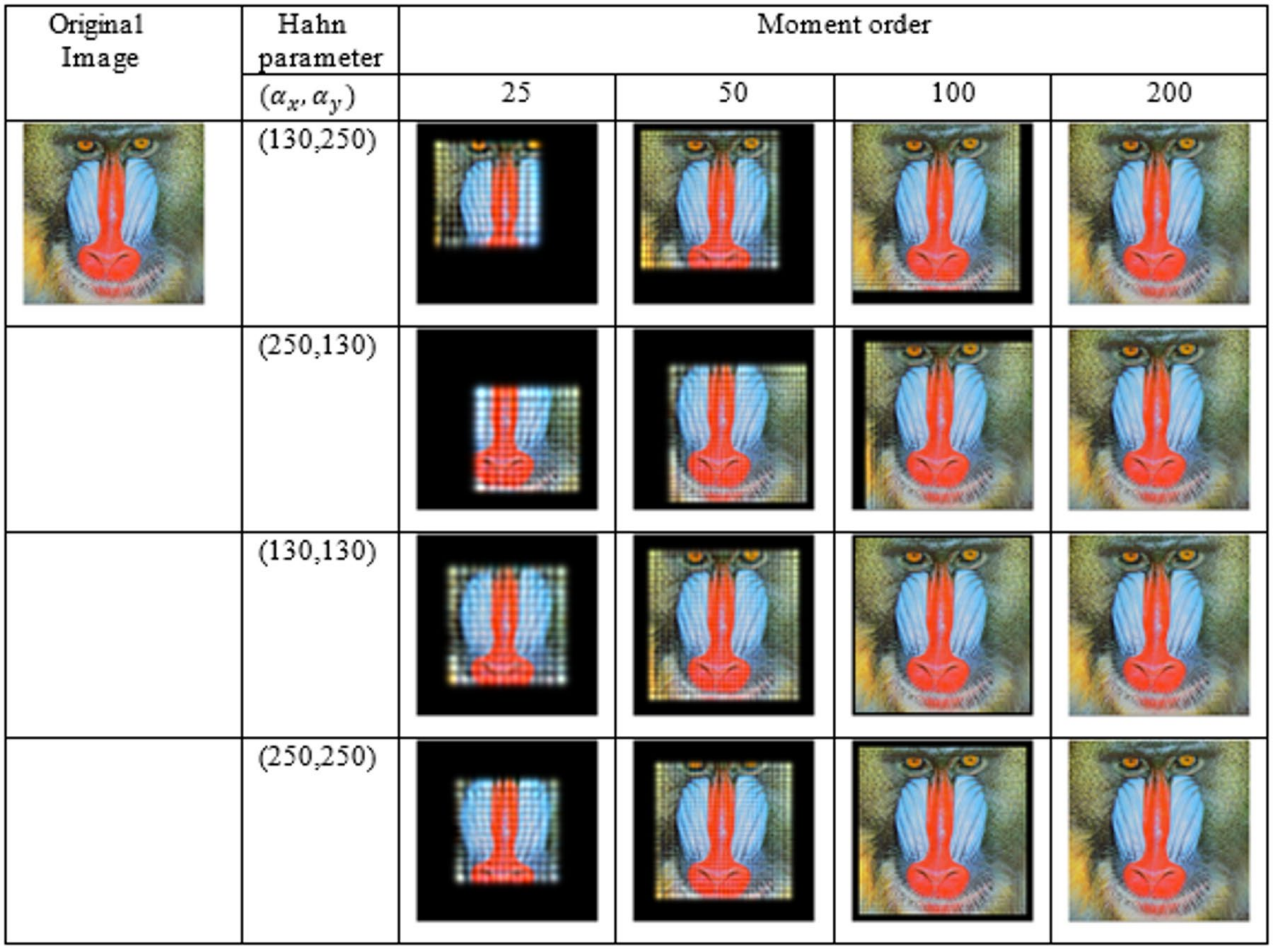
Samples of images reconstructed from the “Mandrill” color image (512 × 512 pixels) using the proposed method. The results are shown for the moment orders of 25, 50, 100, and 200 and transformation parameters $({\boldsymbol{\alpha}}_{\boldsymbol{x}},{\boldsymbol{\alpha}}_{\boldsymbol{y}})$ = (130, 250), (250, 130), (130,130), and (250, 250).

**Fig. 2:**
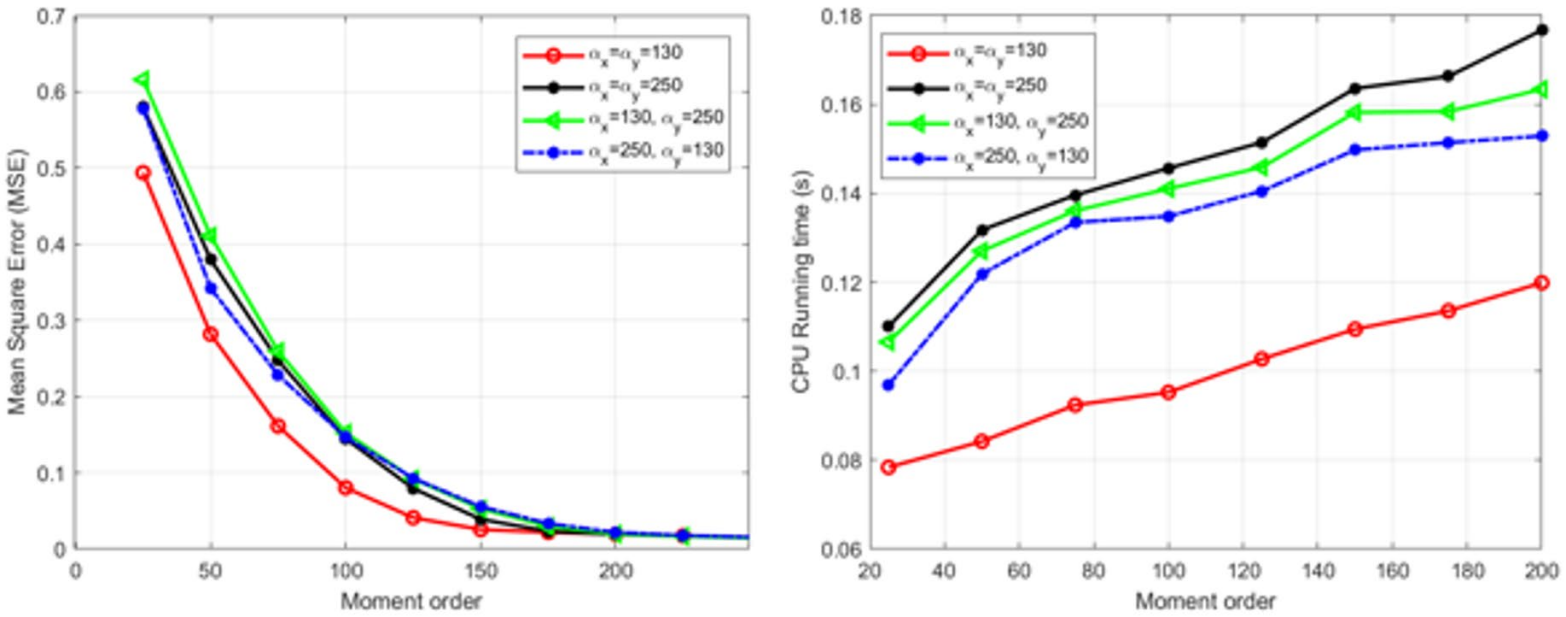
MSE values (left) and CPU runtimes (right) of the proposed reconstruction algorithm on the “Mandrill” color image (512 × 512 pixels), evaluated at moment orders of 25, 50, … 200.

The proposed algorithm is evaluated using the 512 × 512 × 3 ‘Mandrill’ image, which is reconstructed via the recurrence relation given in (13). Reconstructions performed with moment orders ranging from 25 to 200 and Hahn parameters of $({\alpha}_{x},{\alpha}_{y})$ = (130,130), (130,250), (250,130), and (250,250) indicate that higher orders yield finer details and improved accuracy. The visual effects of the moment order and the selected parameters are shown in Fig. 1, with certain combinations producing sharper results. The mean squared error (MSE) and CPU runtime are shown in Fig. 2, which reveals that the MSE decreases as the moment order increases, whereas conducting reconstruction with (130,130) achieves real-time performance (≈ 0.1 s). Overall, the algorithm demonstrates high accuracy and low computational complexity, particularly at a moment order of 200 with parameters of $\left({\alpha}_{x},{\alpha}_{y}\right)$= (130,130).

### Color image watermarking

In image watermarking cases, Hahn moments and Arnold transformations are combined to embed and extract watermarks, ensuring both robustness and imperceptibility. Hahn moments provide robust reconstruction capabilities and maintain orthogonality, whereas the Arnold transform introduces scrambling to provide enhanced security. This integration scheme ensures resilience against common image distortions, while preserving high visual quality. The detailed steps of the proposed embedding algorithm are outlined below.

### Embedding procedure

**Step 1**. To provide enhanced security, the binary watermark is scrambled using the Arnold transform, which eliminates pixel correlation and makes the watermark unrecognizable. This scrambling process provides cryptographic protection since the same keys are required for the inverse reconstruction procedure. Without these keys, attackers cannot restore the original bit order. For an × binary watermark, the permutation is defined as follows: $$\left(\genfrac{}{}{0pt}{}{{i}_{1}}{{j}_{1}}\right)=\left[\left(\begin{array}{cc}1&1\\k&k+1\end{array}\right)\left(\genfrac{}{}{0pt}{}{i}{j}\right)\right]mod\left(n\right)$$ where $(i,j)$ represents the coordinate of the original watermark pixels, $({i}_{1},{j}_{1})$ represents the coordinate of the scrambled watermark pixels, and $k$ and $n$ are the key parameters given by the user.

**Step 2.** In the proposed method, the red channel of the RGB host image $h\left(x,y,k\right)$ is selected for embedding the scrambled watermark $W1$. While the red channel is chosen, the green or blue channels can also be used. The scrambled watermark, which is obtained by the Arnold transform in step 1, is embedded into $h\left(x,y,1\right)$ to protect the host image.

**Step 3**. In this step, forward Hahn moments are applied to the red channel $h(x,y,1)$ of the host image. The image is divided into nonoverlapping $8\times8$ blocks, and for each block, the corresponding Hahn moment coefficient ${\boldsymbol{Q}}_{\mathrm{8,8}}$ is computed using Algorithm 1. Here, ${Q}_{xy}$ denotes the Hahn moment coefficient at position $(x,y)$ within the transformed block. Given the quantization step size $\varDelta$ and having obtained the moment ${\boldsymbol{Q}}_{\mathrm{8,8}}$, we embed the scrambled binary watermark image ${W}_{1}$ (generated in Step 1) into $h(x,y,1)$ using a quantization-based embedding rule:

- If $${W}_{1}\left(i,j\right)=1,$$ then: $${T}_{1}=0.1\times\varDelta,{T}_{2}=-1.1\times\varDelta$$
- If $${W}_{1}\left(i,j\right)=0,$$ then: $${T}_{1}=-0.1\times\varDelta,{T}_{2}=1.1\times\varDelta$$

Then, the quantization candidates are computed as follows: ${C}_{1}={T}_{1}+2R\varDelta$, and ${C}_{2}={T}_{2}+2R\varDelta$, where $R=\lfloor\frac{\lceil{Q}_{\mathrm{8,8}}/\varDelta\rceil}{2}\rfloor$.

Finally, the Hahn moment coefficient ${Q}_{\mathrm{8,8}}$ is modified on the basis of proximity to the quantization candidates:

24 $${Q}_{\mathrm{8,8}}^{{\prime}}=\left\{\begin{array}{c}{C}_{2},\left|{Q}_{\mathrm{8,8}}-{C}_{2}\right|<\left|{Q}_{\mathrm{8,8}}-{C}_{1}\right|\\{C}_{1},otherwise.\end{array}\right.$$

For a $512\times512\times3$ host image, the red channel $h(x,y,1)$is divided into $8\times8$ blocks, yielding an embedding capacity of $(512/8)\times(512/8)=4096$ bits. Thus, the binary watermark must have a size of $64\times64=4096$ to match this capacity.

**Step 4**. After the embedding process, Algorithm 2 is applied to each modified block to reconstruct the red channel ${h}^{*}(x,y,1)$ containing the watermark. This reconstructed channel is then combined with the unchanged green $h(x,y,2)$ and blue $h(x,y,3)$ channels.

### Extraction procedure

The proposed blind watermarking method extracts the watermark without needing the original image but requires the Hahn polynomial and Arnold transform keys used during embedding. The extraction steps starting from the watermarked image ${h}^{*}(x,y,k)$are outlined below.

**Step 1.** The red channel ${h}^{*}(x,y,1)$ is extracted from the color watermark image ${h}^{*}(x,y,k)$.

**Step 2.** ${h}^{*}(x,y,1)$ is divided into nonoverlapping $8\times8$ blocks.

**Step 3.** The Hahn moment coefficients ${Q}_{\mathrm{8,8}}^{*}$ for each block are computed.

**Step 4.** The binary bits of the scrambled watermark ${W}_{1}^{*}$ are extracted using the following function:

25 $${W}_{1}^{\mathrm{*}}(i,j)=\left\{\begin{array}{c}1,if\left|{Q}_{\mathrm{8,8}}^{\mathrm{*}}\right|-2\varDelta\times{round}\left(\frac{{Q}_{\mathrm{8,8}}^{\mathrm{*}}}{2\varDelta}\right)>0\\0,if\left|{Q}_{\mathrm{8,8}}^{\mathrm{*}}\right|-2\varDelta\times{round}\left(\frac{{Q}_{\mathrm{8,8}}^{\mathrm{*}}}{2\varDelta}\right)\le0\end{array}\right.$$

**Step 5.** The inverse Arnold transform is applied to ${W}_{1}^{*}$ to retrieve the final extracted watermark ${W}^{*}$.

**Fig. 3:**
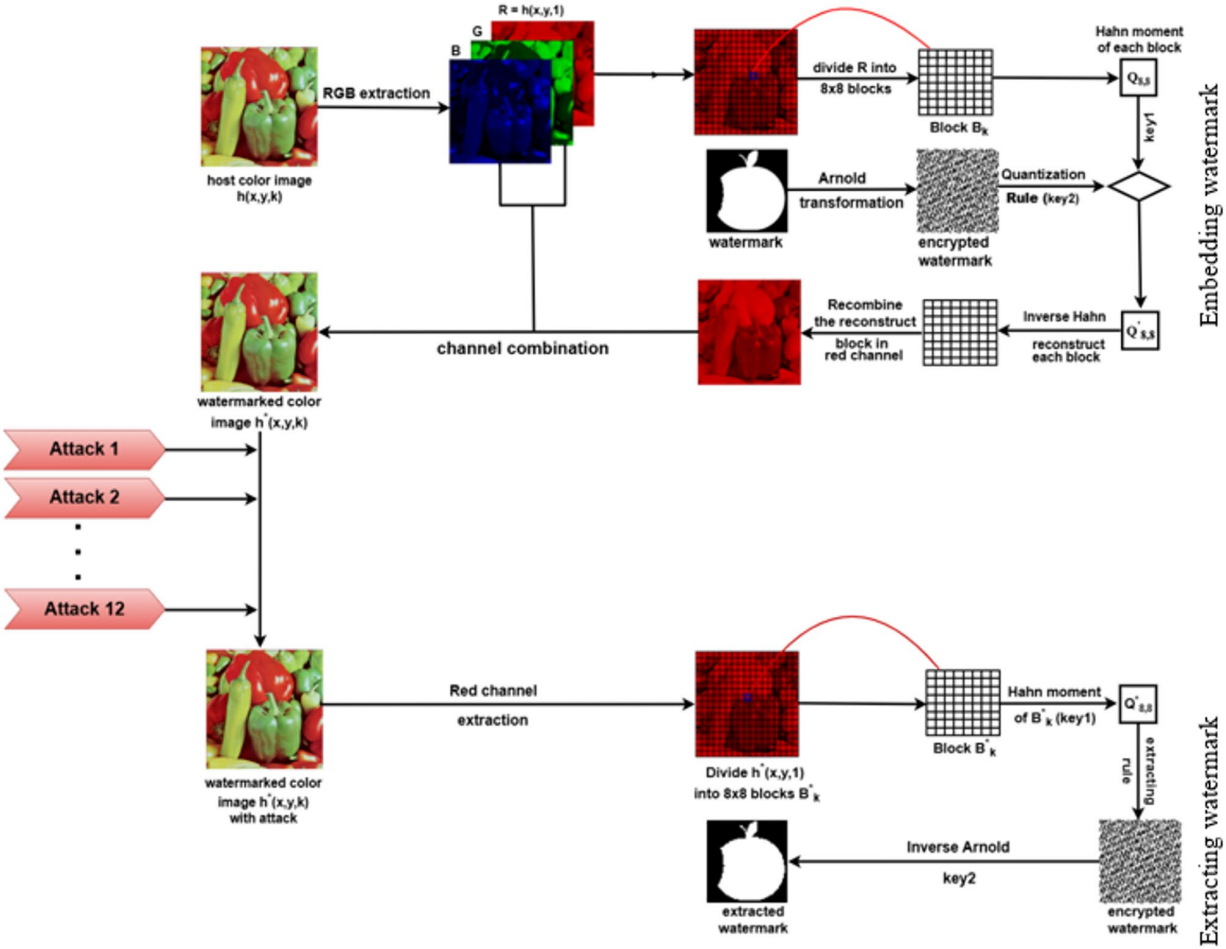
Flowchart of the proposed blind color image watermarking scheme.

A flowchart illustrating the workflow of the proposed technique for embedding and extracting the apple watermark in the color Peppers image is shown in Fig. 3.

## Results and discussion

**Fig. 4:**
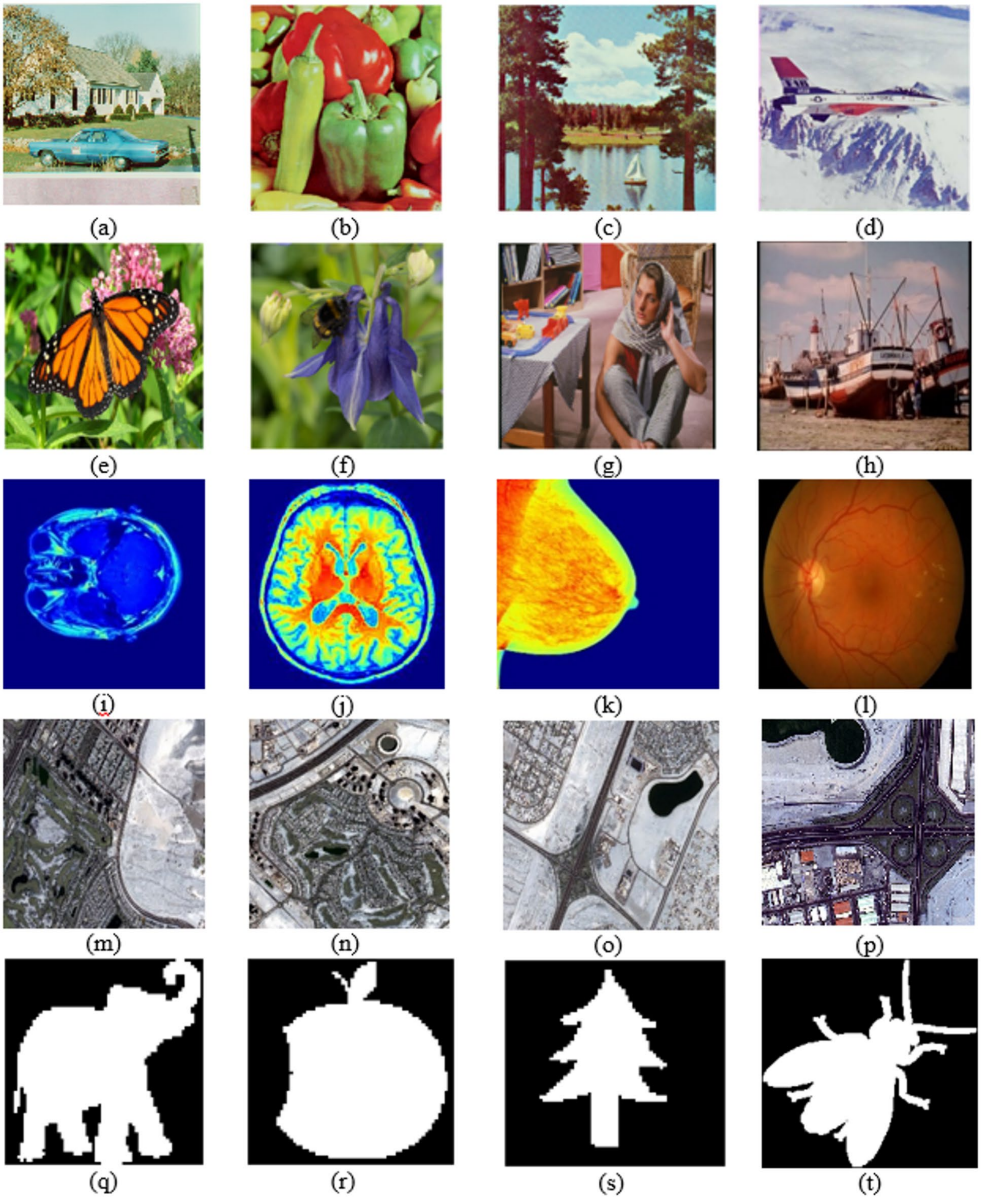
The dataset used in the experiments: natural images (**a–h**), medical images (**i–l**), aerial images (**m–p**), and binary watermark images (**q–t**). (**a**) House, (**b**) Peppers, (**c**) Sailboat, (**d**) Airplane, (**e**) Butterfly, (**f**) Bee, (**g**) Barbara, (**h**) Boats, (**i**) Brain Cancer, (**j**) Brain Tumor, (**k**) Breast Cancer, (**l**) Fundus, (**m**) Aerial 1, (**n**) Aerial 2, (**o**) Aerial 3, (**p**) Aerial 4, (**q**) Elephant, (**r**) Apple, (**s**) Tree, and (**t**) Fly.

To assess the performance of the proposed watermarking algorithm, experiments were conducted on a dataset comprising three main classes of color images that were used as host images, along with four binary watermark images^47^(Figs. 4(q–t)). The size of each color host image was 512 × 512 × 3, while the size of each watermark image was 64 × 64. The first class of the dataset included natural color images^48^ such as House, Peppers, Sailboat, and others, as shown in Figs. 4(a–h). The second class included four medical color images, namely Brain Cancer^49^, Brain Tumor^49^, Breast Cancer^50^, and Fundus^51^, as illustrated in Figs. 4(i–l). The third class included four color aerial images^52^ captured from different viewpoints, as shown in Figs. 4(m–p). Fair comparisons were conducted with five existing watermarking methods^23,33–36^.

### Imperceptibility without attacks

To evaluate the imperceptibility and robustness of the proposed watermarking algorithm, four standard metrics were used: the structural similarity index measurement (SSIM), peak signal-to-noise ratio (PSNR), normalized cross-correlation (NCC), and bit error rate (BER). SSIM and PSNR measure the visual quality of the watermarked image, whereas the NCC and BER assess the robustness of the extracted watermark. SSIM evaluates the similarity between the original image *H* and the watermarked image ${H}^{w}$^30^. When SSIM is approximately 1, the watermarked image is of high quality; when it is near 0, the quality level is poor. It is defined as follows:

26 $$SSIM\left(H,{H}^{w}\right)=\frac{\left(2{\mu}_{H}{\mu}_{{H}^{w}}+{C}_{1}\right)\left(2{\rho}_{H{H}^{w}}+{C}_{2}\right)}{\left({\mu}_{H}^{2}+{\mu}_{{H}^{w}}^{2}+{C}_{1}\right)\left({\sigma}_{H}^{2}+{\sigma}_{{H}^{w}}^{2}+{C}_{2}\right)}$$

where ${\mu}_{H}$, ${\mu}_{{H}^{w}}$, ${\sigma}_{H}^{2}$, ${\sigma}_{{H}^{w}}^{2}$, and ${\rho}_{H{H}^{w}}$ represent the means, variances, and covariance, respectively, of both images. PSNR measures the fidelity of the target image in decibels (dB)^29^. A higher PSNR (> 37 dB) indicates better imperceptibility.

27 $$PSNR=10{log}_{10}\left(\frac{{255}^{2}}{MSE}\right)$$

Where

28 $$MSE=\frac{1}{MN}\sum_{i=0}^{M-1}\sum_{j=0}^{N-1}{\left[H\left(i,j\right)-{H}^{w}(i,j)\right]}^{2}$$

NCC evaluates the similarity between the original watermark $W\left(i,j\right)$ and the extracted watermark ${W}^{*}(i,j)$^31^. Values close to 1 indicate stronger robustness:

29 $$NCC=\frac{\sum_{i=0}^{M}\sum_{j=0}^{N}\left[W\left(i,j\right)\times{W}^{\mathrm{*}}(i,j)\right]}{\sum_{i=0}^{M}\sum_{j=0}^{N}{\left[W\left(i,j\right)\right]}^{2}}$$

BER is the ratio of the number of incorrectly extracted bits to the total number of embedded bits^32^:

30 $$BER=\frac{\sum_{i=0}^{M-1}\sum_{j=0}^{N-1}\left[W\left(i,j\right)\oplus{W}^{\mathrm{*}}(i,j)\right]}{M\times{N}}$$

A lower BER indicates greater robustness against attacks.

**Fig. 5:**
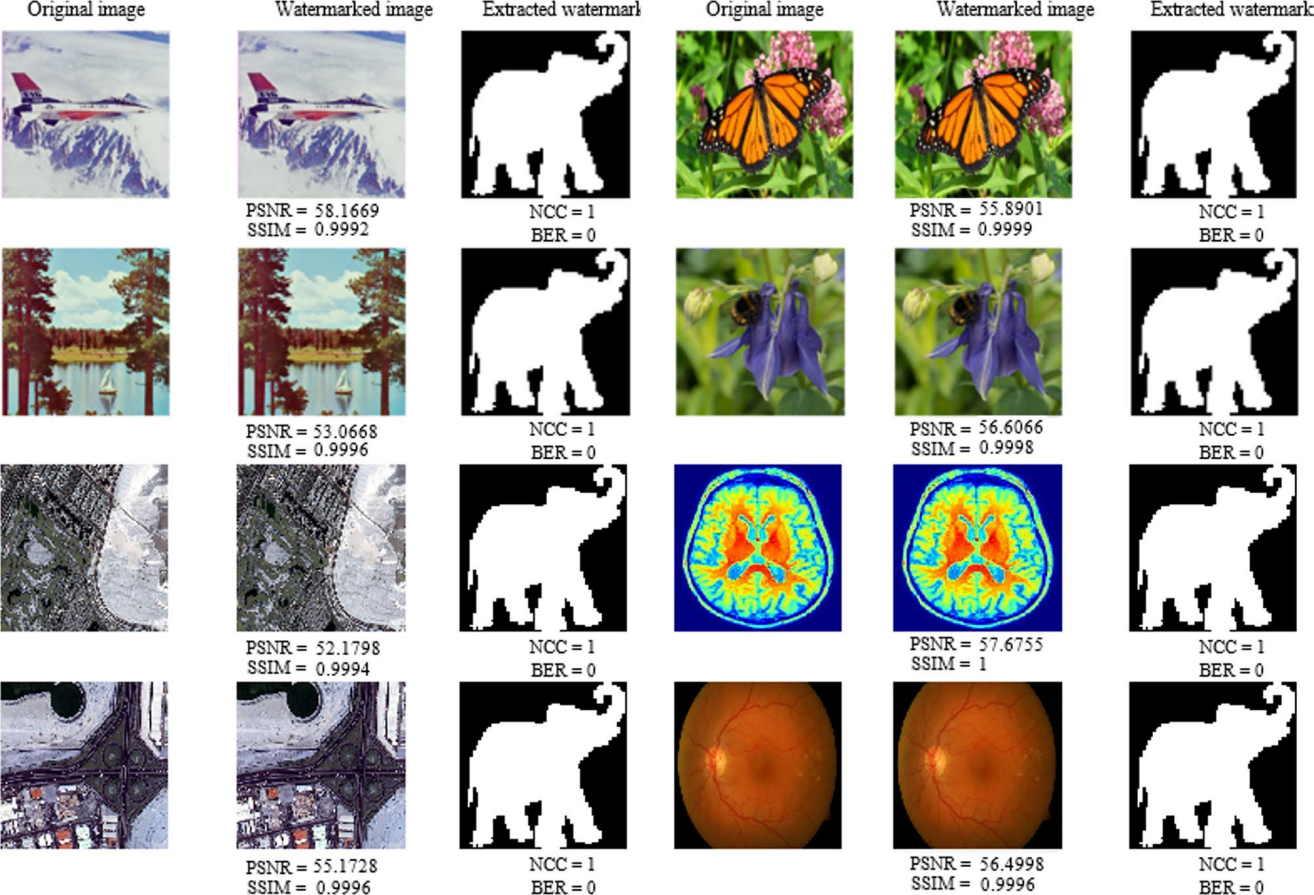
Embedding and extraction results obtained for the elephant watermark by applying the proposed method to the entire dataset under no-attack conditions.

**Fig. 6:**
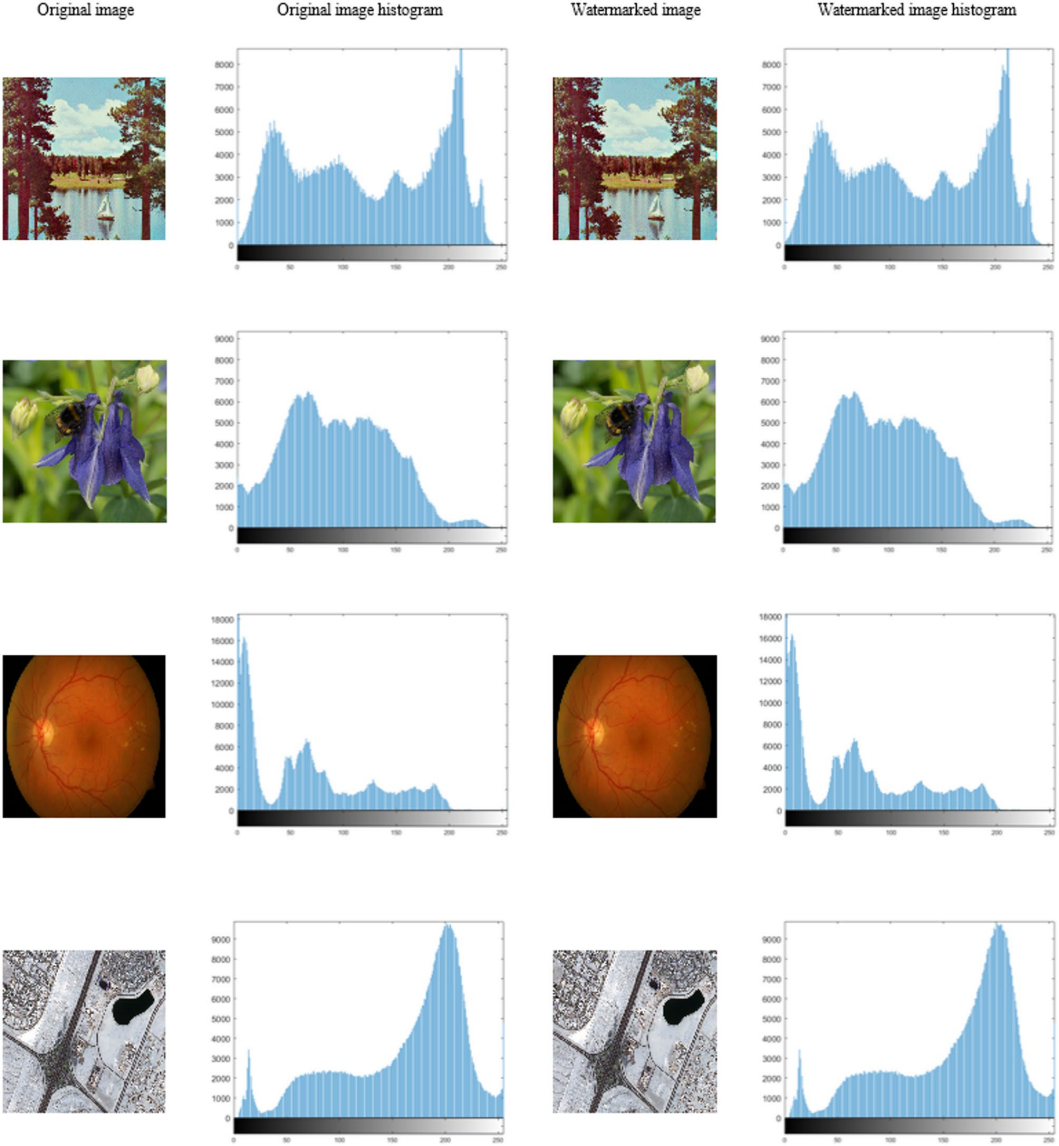
Histograms of the original host image and the corresponding watermarked image for each sample from the dataset.

In the first experiment, the proposed algorithm, with a quantization step size of ∆ = 45, was applied to all the images contained in the dataset using the elephant watermark. The results in Fig. 5 indicate that the watermarked images closely resemble the originals, with their SSIM values approaching 1. NCC = 1 and BER = 0 confirm the excellent robustness and imperceptibility of the method. Figure 6 displays the histograms of the original host image and the corresponding watermarked image for each class in the dataset. The results indicate that the histograms of both images are identical, indicating that the embedding process introduced no significant distortion in the statistical distribution of the pixel intensities. This high degree of histogram similarity demonstrates that the proposed method preserved both the visual quality and statistical characteristics of the host image, making the embedded watermark imperceptible to the human visual system.

**Fig. 7:**
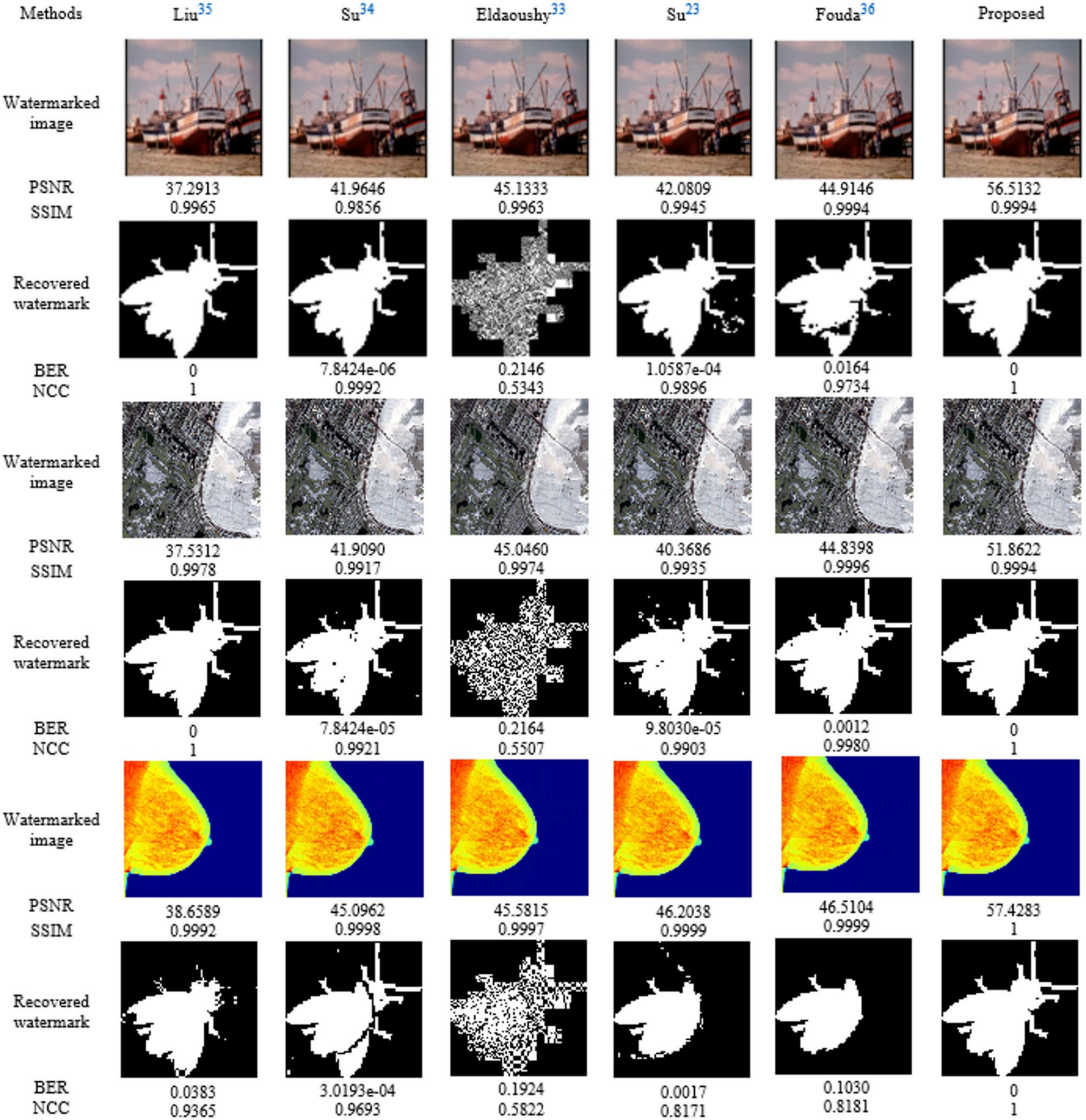
Imperceptibility performance: watermarked image and corresponding recovered watermarks produced with various algorithms and their associated PSNRs, SSIMs, BERs, and NCCs values for representative samples from the dataset.

To further verify the imperceptibility of the watermark, five existing techniques^23,33–36^ were used for comparison purposes. The watermarked and extracted images produced for the natural Boats, Aerial 1, and medical Breast Cancer host images when ∆ = 50 with a fly watermark are shown in Fig. 7 The proposed method achieved high imperceptibility, particularly for medical images (PSNR = 57.4283 dB and SSIM = 1), along with perfect watermark recovery (BER = 0). In contrast, the other methods resulted in lower imperceptibility and higher BER values. Overall, the proposed algorithm outperformed the other methods in terms of imperceptibility and robustness.

**Fig. 8:**
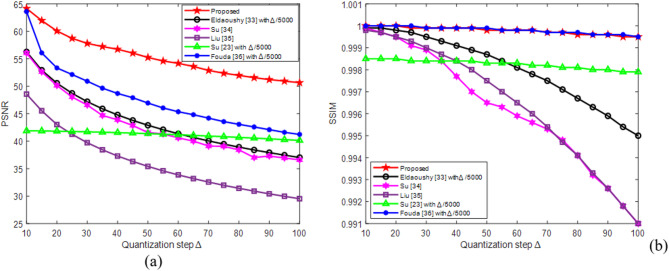
PSNR and SSIM values produced for the boats watermarked image with different quantization step sizes for various watermarking algorithms.

To evaluate the impact of the quantization step on the considered methods, the apple watermark was extracted from the Butterfly host image using step sizes Δ ranging from 10 to 100. The data in Fig. 8(a) and (b) indicate that PSNR and SSIM values decreased as ∆ increased. At ∆ = 50, the proposed algorithm achieved PSNR of 55.2429 and SSIM of 0.9998, surpassing the results of the other methods^23,33–36^. The competing algorithms exhibited lower PSNR values (35.4130–46.9420) and SSIM values (0.9965–0.9999). Thus, the proposed method demonstrates superior watermark imperceptibility under the same conditions.

**Fig. 9:**
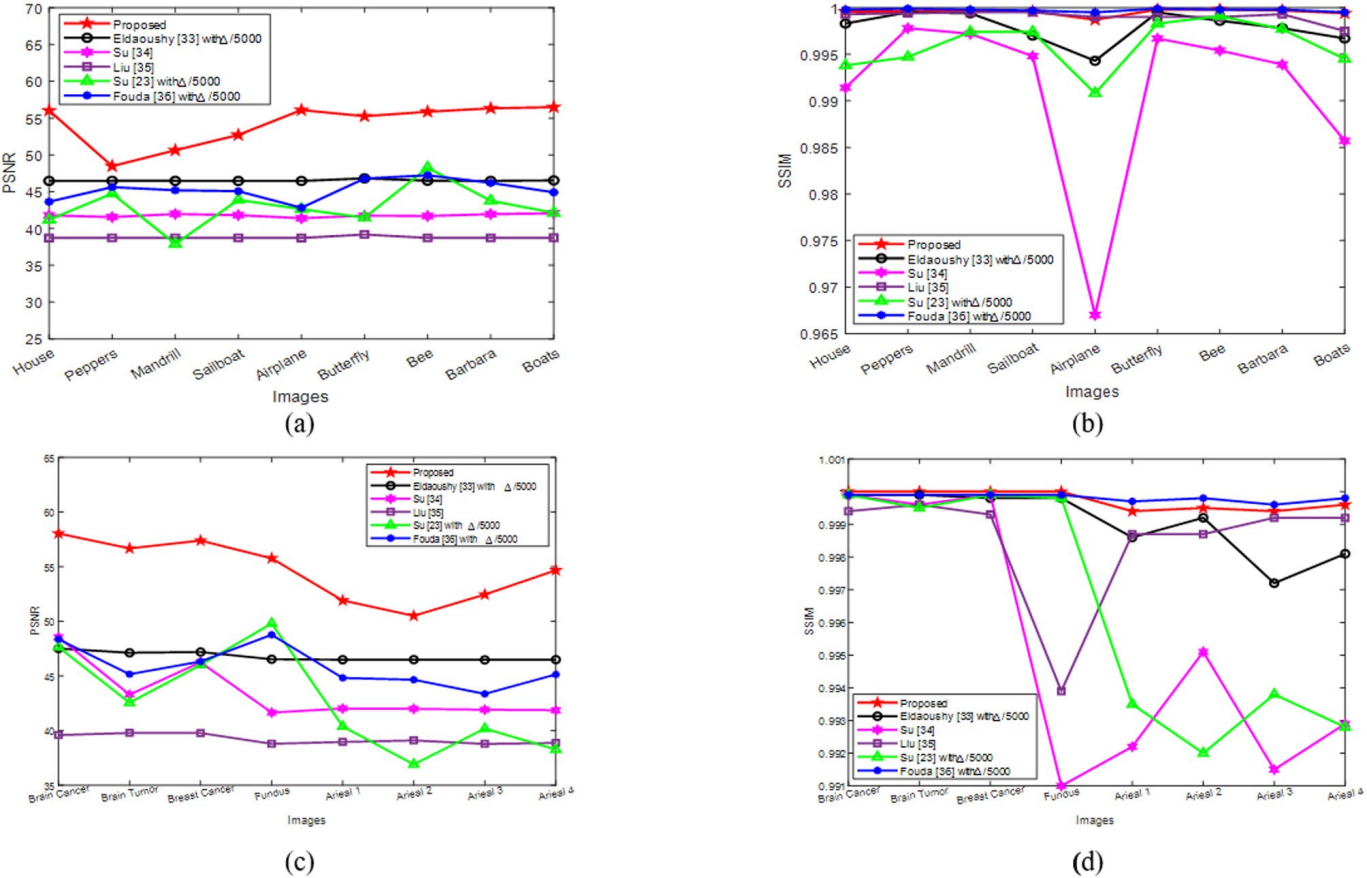
Comparison among the PSNR and SSIM values of different methods: (**a**) PSNRs achieved for watermarked natural images, (**b**) SSIMs attained for watermarked natural images, (**c**) PSNRs produced for watermarked medical and aerial images, and (**d**) SSIMs of the watermarked medical and aerial images.

All color host images acquired from the three dataset classes, along with the Tree watermark, were used to compare PSNR and SSIM values attained under no-attack conditions with Δ = 50. The results in Figs. 9(a, c) show that Liu^35^ achieved the lowest PSNR values for natural, medical, and aerial images, except for the Aerial 2 image. Moreover, the results displayed in Figs. 9(b, d) indicate that Su^34^ obtained the lowest SSIM values for all the images across the three classes, except for the Aerial 2 image. Furthermore, the results presented in Figs. 9(a–d) demonstrate that the proposed algorithm achieved the highest PSNR and SSIM values among all the compared methods for all the images in the three classes. Consequently, the imperceptibility of the proposed method is superior to those of the other algorithms.

**Fig. 10:**
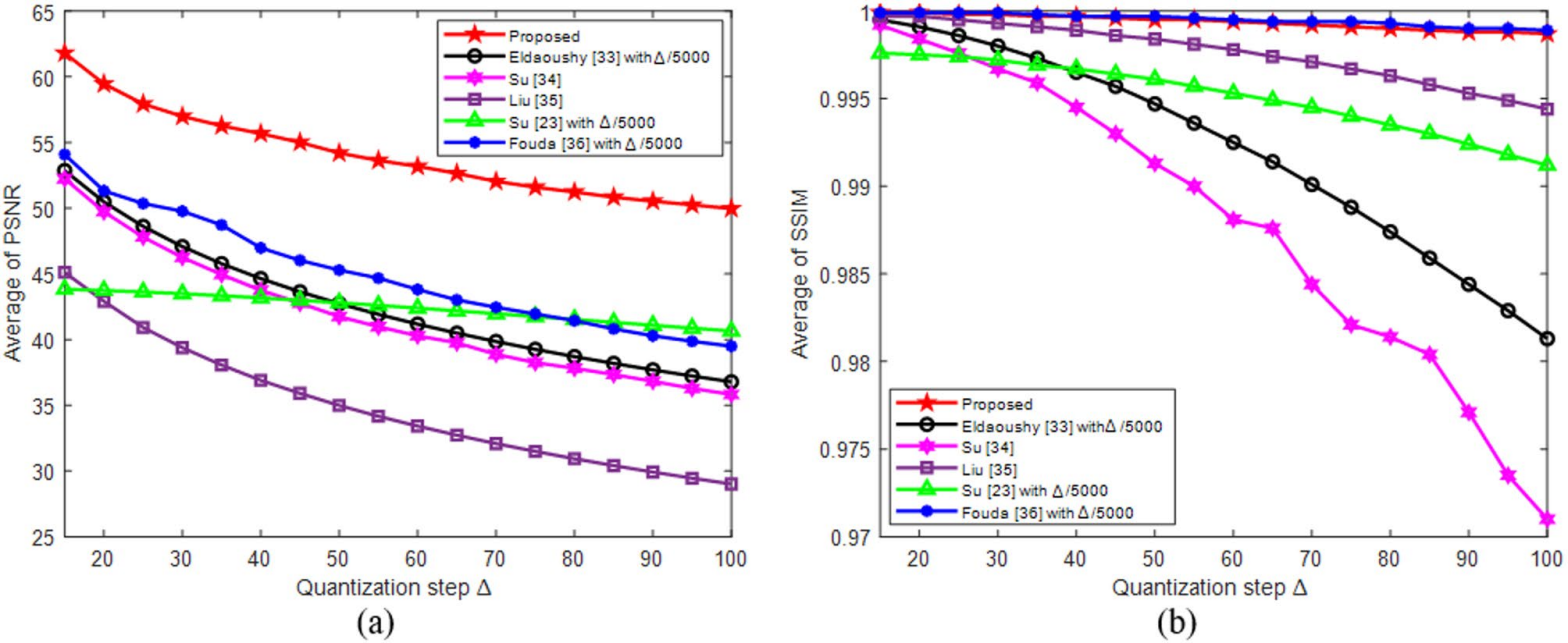
Averages of PSNR and SSIM values produced for the nine watermarked images with different quantization step sizes for various watermarking algorithms.

The average PSNR and SSIM values yielded by the proposed and compared algorithms were analyzed across different quantization steps sizes Δ, as illustrated in Fig. 10. PSNR values generally decreased with increasing Δ; however, the proposed algorithm consistently maintained significantly higher PSNR values (> 60 dB), far exceeding the imperceptibility threshold of 37 dB. This demonstrates its robust ability to preserve image quality while maintaining robustness against attacks. Similarly, SSIM values in Fig. 10(b) remain close to 1 for the proposed algorithm and Fouda^36^, indicating excellent structural preservation. Other methods, particularly Su^34^ and Eldaoushy^33^, showed sharp decreases in their SSIMs as Δ increased. Thus, the proposed algorithm maintained superior image fidelity across all the Δ values.

It effectively balances imperceptibility and robustness, consistently outperforming all the compared techniques.

All color host images acquired from the three dataset classes, along with the Tree watermark, were used to evaluate the proposed algorithm against the methods reported in^23,33–36^ using NCC and BER metrics at Δ = 50. Tables 2 and 3 present the NCC and BER values obtained for representative samples from the three classes. The results in these tables indicate that the proposed algorithm consistently achieved perfect performance (NCC = 1 and BER = 0), demonstrating flawless watermark recovery capabilities. Liu^35^ reported comparable performance, while Su^34^ also yielded strong results. In contrast, Eldaoushy^33^ performed poorly, yielding low NCC values (0.51–0.54) and high BER values (≈ 0.15) for natural images. Su^23^ achieved moderate performance but failed to reach perfect results for both metrics. Consequently, the proposed watermarking algorithm demonstrated superior robustness and accuracy across all the tested images.

**Table 2:** Performance comparison between the proposed method and other techniques in terms of NCC metrics for representative samples derived from the three dataset classes.

Images	Liu^35^	Su^34^	Su^23^ (∆/5000)	Eldaoushy^33^ (∆/5000)	Fouda^36^ (∆/5000)	Proposed
Airplane	1	1	0.8755	0.5433	0.9983	1
Butterfly	1	0.9878	0.9776	0.5138	0.9839	1
Bee	1	0.9944	0.9961	0.5307	0.9681	1
Boats	1	1	0.9859	0.5347	0.9771	1
Brain Cancer	1	0.5283	0.4383	0.5256	0.3697	1
Breast Cancer	0.9692	0.9559	0.5675	0.8598	0.8620	1
Arieal 1	1	0.9883	0.5452	0.9874	0.9983	1
Arieal 4	1	0.9833	0.5386	0.9771	0.9872	0.9994

**Table 3:** Performance comparison between the proposed method and other techniques in terms of BER metrics for representative samples derived from the three dataset classes.

Images	Liu^35^	Su^34^	Su^23^ (∆/5000)	Eldaoushy^33^ (∆/5000)	Fouda^36^ (∆/5000)	Proposed
Airplane	0	0	9.9598e-04	0.1557	7.3242e-04	0
Butterfly	0	8.6266e-05	1.6077e-04	0.1555	0.0071	0
Bee	0	3.9212e-05	2.7448e-05	0.1536	0.0139	0
Boats	0	0	1.0195e-04	0.1523	0.0100	0
Brain Cancer	0	0.0026	0.1711	0.0032	0.1912	0
Breast Cancer	0.0134	3.0585E-04	0.1396	9.2540E-04	0.0569	0
Arieal 1	0	8.2345E-05	0.1551	9.0187E-05	7.3242e-04	0
Arieal 4	0	1.1764E-04	0.1538	1.6469E-04	0.0056	3.9212E-06

### Watermarking attacks

Once imperceptibility was ensured, the robustness of the method was evaluated by testing the resistance of the watermark to various attacks. In the context of watermarking, robustness refers to the ability to accurately extract the watermark after distortions occur. The proposed technique was evaluated against noise, filtering, geometric, and robustness attacks using BER and NCC metrics and was compared with recently proposed state-of-the-art methods.

### Noise attacks

**Fig. 11:**
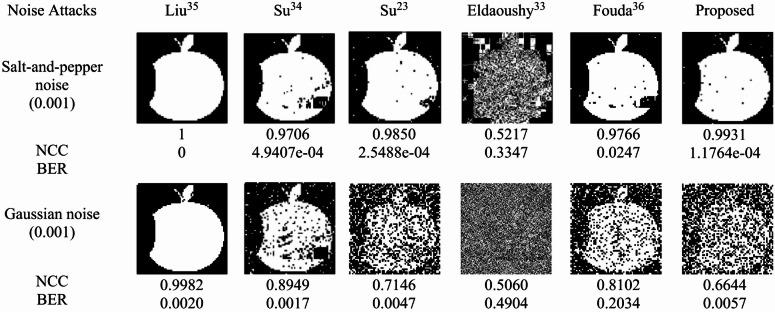
Extracted watermarks and the corresponding NCC and BER values produced under noise attacks.

Noise attacks evaluate the robustness of watermarking methods against random image brightness or color variations. Two types of noise, namely, salt-and-pepper (density 0.001) and Gaussian noise (variance 0.001), are added to the watermarked image. The data in Fig. 11 indicate that both Liu^35^ and the proposed method performed best under salt-and-pepper noise. However, under Gaussian noise, the proposed method still detected the watermark, although the extracted image was slightly degraded.

### Filter attacks

**Fig. 12:**
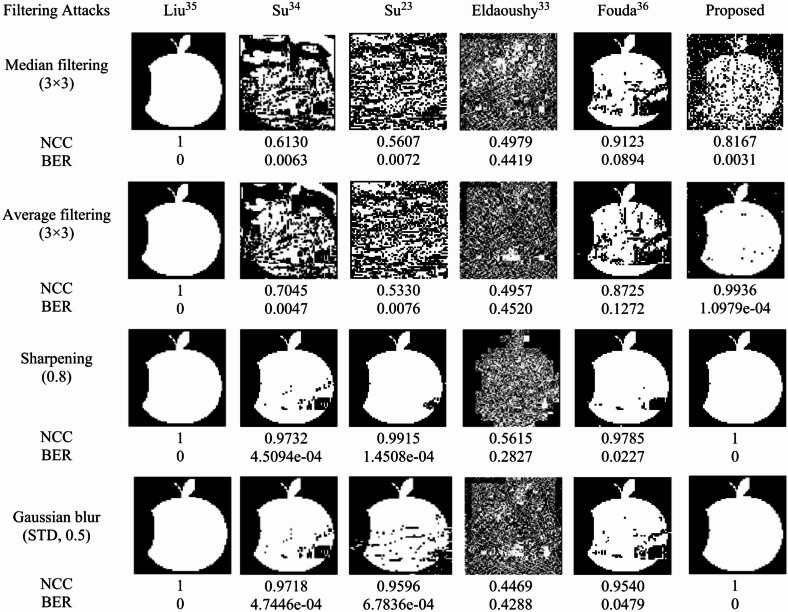
Extracted watermarks and the corresponding NCC and BER values produced under filtering attacks.

Filter attacks are classified into four types: median, average, sharpening, and blurring attacks. These operations modify or enhance images and are used to evaluate the robustness of watermarking algorithms. The proposed method, along with other algorithms, was evaluated using the “Boats” image under these attacks. The results in Fig. 12 indicate that for the median filter, Liu^35^ and Fouda^36^ performed best, whereas the proposed method ranked second in terms of BER. Under the average filter, the proposed algorithm again ranked second, outperforming most of the competing methods. For sharpening (0.8) and blurring (0.5) attacks, Su^34^, Su^23^, and Eldaoushy^33^ produced degraded watermarks, whereas the proposed method and Liu^35^ perfectly recovered the watermark, achieving BER = 0 and NCC = 1. These results confirm that the proposed algorithm maintains high robustness against major filtering attacks.

### Robustness attacks

**Fig. 13:**
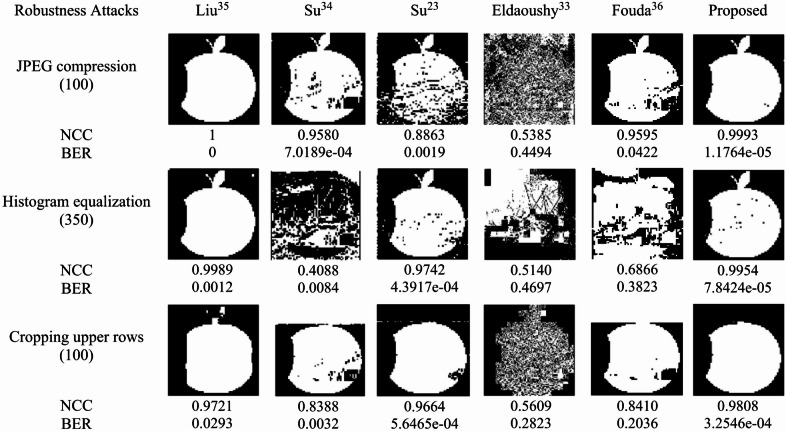
Extracted watermarks and the corresponding NCC and BER values produced under robustness attacks.

Robustness attacks include JPEG compression, histogram equalization, and cropping. Under JPEG compression (quality factor = 100), the second row of Fig. 13 illustrates that the proposed and Liu^35^ methods produced high-quality extracted watermarks with excellent NCC and BER values, confirming their strong robustness. With respect to histogram equalization (350 bins), both methods achieved the best results from visual and numerical perspectives, as shown in the middle row of the figure. In the cropping test, 100 pixels were removed from the upper part of the “Boats” image. The last row in Fig. 13 displays the extracted watermarks along with the corresponding robustness metrics. The proposed method achieved the highest NCC (0.9808) and the lowest BER (3.25 × 10⁻⁴), demonstrating its superior resilience against content losses and the highest overall robustness.

### Geometric attacks

**Fig. 14:**
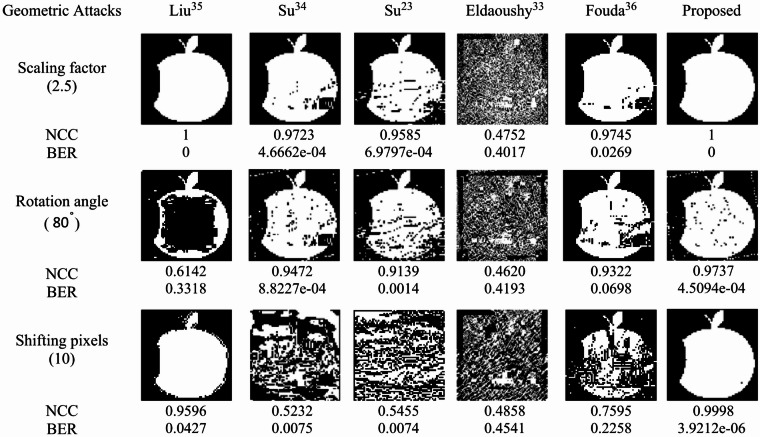
Extracted watermarks and the corresponding NCC and BER values produced under geometric attacks.

Geometric attacks include scaling, rotation, and shifting. In the scaling test, with a magnification factor of 2.5, the proposed watermarking algorithm achieved perfect results (NCC = 1, BER = 0), confirming its strong robustness. Under rotation attacks, the given images were rotated at various angles and then counter-rotated for recovery; the proposed method demonstrated superior performance, particularly at 80°, 90°, and 100°. For shifting attacks, the image was horizontally moved by 10, 50, and 100 pixels. As shown in Fig. 14, the proposed method consistently achieved the highest NCC values, demonstrating strong resistance to geometric distortions.

**Fig. 15:**
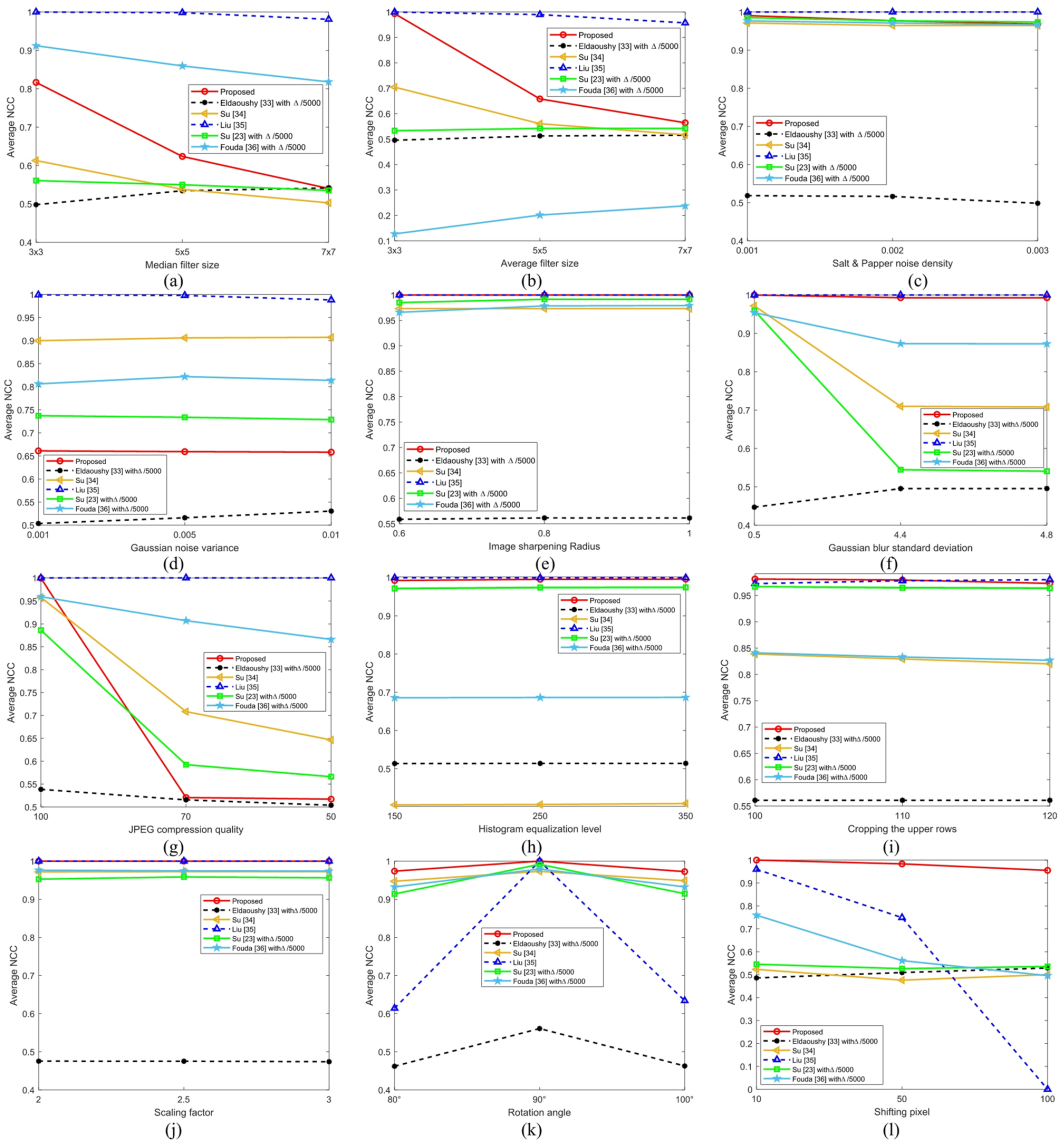
The average NCC values of the watermarks extracted under different attacks: (**a**) median filtering, (**b**) average filtering, (**c**) salt-and-pepper noise, (**d**) gaussian noise, (**e**) sharpening, (**f**) gaussian blur, (**g**) JPEG compression, (**h**) histogram equalization, (**i**) cropping (**j**) rescaling, (**k**) rotation, and (**l**) shifting.

To evaluate the robustness of the proposed algorithm, the average NCC values were calculated under twelve different types of attacks. The performance of the proposed method was compared with that of five state-of-the-art methods across three parameter levels for each type of attack in Fig. 15. The proposed Hahn moment-based algorithm demonstrated strong robustness against most attacks, such as sharpening, blurring, scaling, and rotation, with NCC values approaching 1. However, its performance slightly decreased under median filtering, Gaussian noise, and JPEG compression attacks. Overall, the proposed method outperformed the existing techniques, ensuring both imperceptibility and robustness.

The robustness of the proposed method was further evaluated using the BER metric across twelve different types of attacks. Figures 11, 12, 13 and 14 illustrate visual comparisons between the extracted apple watermark and those of the five other methods. Table 4 presents the average BER values produced across three parameter levels for each type of attack. The proposed algorithm and Liu^35^ achieved near-zero BERs for most attacks, indicating strong robustness. Overall, the proposed method exhibited superior performance across most attack scenarios, particularly under filtering, sharpening, scaling, and geometric distortions.

**Table 4:** Average BER values produced under twelve attacks for the embedded and extracted apple watermark using the proposed and compared methods.

Attacks	Parameter	Su^34^	Liu^35^	Su^23^	Eldaoushy^33^	Fouda^36^	Proposed
Median filtering	3 **×** 3 5 **×** 5 7 **×** 7	0.0063 0.0073 0.0078	**0** **0.0017** 0.0203	0.0072 0.0073 **0.0075**	0.4419 0.4533 0.4594	0.0894 0.1392 0.1770	0.0031 0.0062 0.0076
Average filtering	3 **×** 3 5 **×** 5 7 **×** 7	0.0047 0.0069 0.0076	**0** 0.0107 0.0452	0.0076 0.0075 0.0075	0.4520 0.4573 0.4527	0.1272 0.2012 0.2375	1.0979E-04 **0.0057** **0.0072**
Salt-and-pepper noise	*d =* 0.001 *d =* 0.002 *d =* 0.003	4.8231E-04 5.9602E-04 5.9210E-04	**0** **0** **0**	2.4703E-04 3.8820E-04 4.4701E-04	0.3245 0.3338 0.3770	0.0247 0.0303 0.0352	1.6077E-04 3.8820E-04 5.4504E-04
Gaussian noise	${\sigma}^{2}=$ 0.001 ${\sigma}^{2}=$ 0.005 ${\sigma}^{2}=$ 0.01	0.0016 **0.0016** **0.0015**	**9.7656E-04** 0.0022 0.0129	0.0044 0.0044 0.0045	0.4927 0.4902 0.4889	0.2056 0.2039 0.2405	0.0057 0.0058 0.0057
Sharpening	0.6 0.8 1	4.5094E-04 4.5094E-04 4.5094E-04	**0** **0** **0**	2.5488E-04 1.4508E-04 1.4508E-04	0.2883 0.2827 0.2822	0.0361 0.0227 0.0220	3.9212E-06 **0** **0**
Gaussian blur	$\sigma=$ 0.5 $\sigma=$ 4.4 $\sigma=$ 4.8	4.7446E-04 0.0046 0.0046	**0** **0** **0**	6.7836E-04 0.0074 0.0075	0.4288 0.4537 0.4523	0.0479 0.1267 0.1270	**0** 1.2156E-04 1.2156E-04
JPEG compression	*q =* 50 *q =* 70 *q =* 100	0.0057 0.0047 7.0189E-04	**0** **0** **0**	0.0069 0.0066 0.0019	0.4667 0.4510 0.4494	0.1338 0.0945 0.0422	0.0080 0.0080 1.1764E-05
Histogram equalization	150 250 350	0.0085 0.0084 0.0084	0.0012 0.0012 0.0012	4.8623E-04 4.4701E-04 4.3917E-04	0.4706 0.4699 0.4697	0.3838 0.3828 0.3823	**1.3724E-04** **8.6266E-05** **7.8424E-05**
Cropping upper rows	100 110 120	0.0032 0.0035 0.0038	0.0293 0.0237 0.0215	5.6465E-04 5.9602E-04 6.0778E-04	0.2823 0.2823 0.2823	0.2036 0.2168 0.2273	**3.2546E-04** **3.5683E-04** **4.6270E-04**
Scaling	2 2.5 3	4.7446E-04 4.6662E-04 4.6662E-04	**0** **0** **0**	7.9208E-04 6.9797E-04 7.3326E-04	0.4102 0.4017 0.3956	0.0251 0.0269 0.0276	**0** **0** **0**
Rotation	${\uptheta}=80^\circ$ ${\uptheta}=90^\circ$ ${\uptheta}=100^\circ$	8.8227E-04 4.5094E-04 8.5874E-04	0.3318 **0** 0.3186	0.0014 1.4508E-04 0.0014	0.4193 0.2823 0.4206	0.0698 0.0220 0.0696	**4.5094E-04** **0** **4.7054E-04**
Shifting	10 50 100	0.0075 0.0082 0.0078	0.0427 0.2554 0.5977	0.0074 0.0077 0.0074	0.4541 0.4399 0.4129	0.2258 0.3921 0.4578	**3.9212E-06** **2.8625E-04** **7.8032E-04**

### Multiple attacks

In addition to demonstrating robustness against individual attacks, the robustness of the proposed watermarking technique was also evaluated under multiple combined attacks that were applied sequentially to the color image dataset. The obtained results are summarized as follows.

- Average filtering and sharpening: The contrast of the watermarked House image was increased by 80%, after which the enhanced image was filtered using a 3 × 3 average filter.
- Rotation and histogram equalization: The watermarked image was first rotated by 80° in the counterclockwise direction, followed by histogram equalization with 350 Gy levels.
- Shifting and scaling: The watermarked image was shifted by 10 pixels in the horizontal direction, and the resulting image was then scaled by a factor of two.
- Salt-and-pepper noise and JPEG compression: The watermarked House image was corrupted with salt-and-pepper noise at a noise density of 0.001 and subsequently compressed using JPEG compression with a quality factor of 100.

**Fig. 16:**
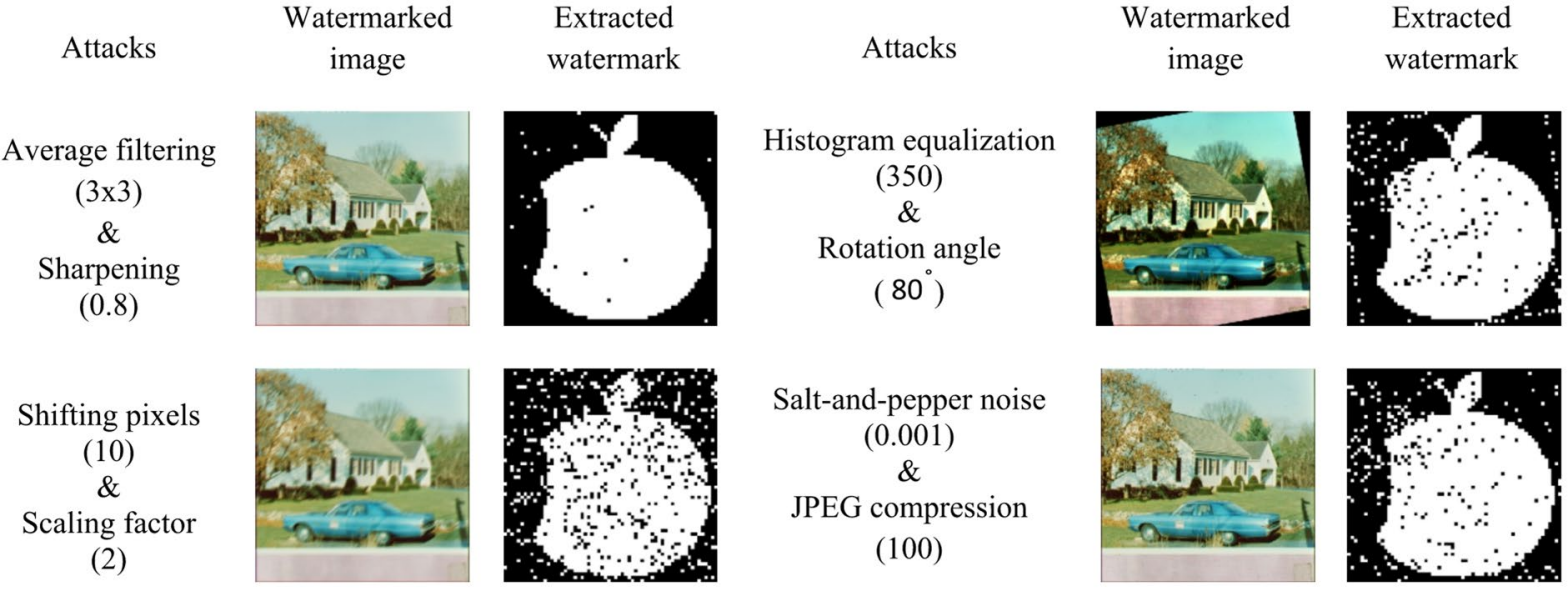
Watermarked images and their corresponding extracted watermarks are subjected to combined attacks.

The resulting images produced under these combined attacks are illustrated in Fig. 16. From the figure, it can be observed that the embedded apple watermark is clearly recognizable even after the application of two simultaneous processing operations. To further evaluate the robustness of the method, various combinations of attacks were applied. Table 5 presents the corresponding BER and NCC values obtained for the extracted watermarks. The results indicate that the proposed watermarking algorithm is highly resilient to multiple combined attacks, as evidenced by the reported quantitative metrics.

**Table 5:** BER and NCC values produced for the extracted watermarks under combined attacks.

Combined attacks	Parameters	BER	NCC
Salt-and-pepper noise & Gaussian noise	*d =* 0.002 & ${\sigma}^{2}=$ 0.001	0.0056	0.6651
Average filtering & Sharpening	3 **×** 3 & 1	9.8030E-05	0.9943
Salt-and-pepper noise & JPEG compression	*d =* 0.001 & *q =* 100	9.4893E-04	0.9445
Gaussian noise & Cropping upper rows	${\sigma}^{2}=$ 0.001 & 100	0.0047	0.7049
Gaussian blur & Median filtering	$\sigma=$ 0.5 & 3 **×** 3	0.0044	0.7285
Histogram equalization & Rotation	350 & ${\uptheta}=80^\circ$	7.8032E-04	0.9543
Average filtering & Rotation	3 **×** 3 & ${\uptheta}=80^\circ$	0.0051	0.6858
Shifting & Scaling	10 & 2	0.0019	0.8885

### Complexity analysis

All the experiments were performed using MATLAB R2024b on a Windows 10 workstation equipped with an Intel^®^ Core™ i7-8550U CPU and 8 GB of RAM. The reported execution times represent the averages measured over the test dataset using the tic/toc functions in MATLAB. The average time required to embed a 64 × 64 watermark into a 512 × 512 × 3 color host image was 0.686168 s per image, whereas the average time needed to extract the same watermark from a watermarked image was 0.289557 s per image. Consequently, the average end-to-end processing time (embedding + extraction) was 0.975725 s per image. Table 6 compares the embedding time, extraction time, and total computational time of the proposed method with those of several existing approaches. The results indicate that the proposed method achieved a total execution time of 0.9757 s, which was comparable to that of Liu^35^ and remained significantly lower than that of Fouda^36^, which required 6.1999 s. Although Su^34^ and Eldaoushy^33^ exhibited lower execution times, the proposed scheme maintained a reasonable computational cost while preserving high robustness and imperceptibility. These results demonstrate that the proposed method provides an effective tradeoff between computational efficiency and watermarking performance.

**Table 6:** Computational time comparison between the proposed method and the existing watermarking schemes in terms of their embedding, extraction, and total execution times.

Methods	Embedding time	Extracting time	Total time
Liu^35^	0.802630	0.086696	0.889326
Su^34^	0.169251	**0.033402**	0.202653
Eldaoushy^33^	**0.122998**	0.078154	**0.201152**
Su^23^	0.312190	0.072105	0.384295
Fouda^36^	4.589398	1.610583	6.199981
Proposed	0.686168	0.289557	0.975725

## Conclusion

A blind watermarking and reconstruction framework for color images in the transformed domain based on Hahn moments is presented in this paper. The method employs an enhanced recurrence relation involving Hahn polynomials to efficiently compute color channel moments (Algorithm 1), followed by a dedicated reconstruction process that accurately reconstructs the original color image from these moments (Algorithm 2). For watermark embedding and extraction purposes, Hahn moments and their inverse transformations are utilized to enable robust and imperceptible watermarking without requiring the original image, ensuring a fully blind and secure scheme. The security of the method is further enhanced by applying Arnold transform into the watermark prior to the embedding stage. Overall, the proposed approach provides a practical copyright protection and ownership verification solution for color images.

Comprehensive experiments conducted on a dataset comprising three types of color images (natural, medical, and aerial images) used as host images, along with four binary watermark images, demonstrate that the proposed scheme outperforms the existing methods. Its high PSNR and SSIM values confirm their excellent visual quality, whereas NCC and BER results indicate strong watermark recovery accuracy. The method further demonstrates notable robustness against a wide range of image processing and geometric attacks, including compression, histogram equalization, cropping, rotation, and salt-and-pepper noise. Despite these strengths, the scheme remains sensitive to median filtering and Gaussian noise attacks. Future work will focus on integrating Hahn moments with additional discrete moment techniques to further improve its robustness under these challenging attack scenarios.

## Data Availability

The codes and data that support the findings of this study are publicly available at the following GitHub repository: https://github.com/melgayar90/Watermarking_Rreconstruction_Using_Hah.

## Code availability

The codes and data that support the findings of this study are publicly available at the following GitHub repository: https://github.com/melgayar90/Watermarking_Rreconstruction_Using_Hah.

## References

[1] Sun, W., Zhou, J., Li, Y., Cheung, M. & She, J. Robust high-capacity watermarking over online social network shared images. *IEEE Trans. Circuits Syst. Video Technol.* **31**(3), 1208–1221. 10.1109/TCSVT.2020.2998476 (2021).

[2] Fang, D. & Sun, S. A new scheme for image steganography based on hyperchaotic map and DNA sequence. *J. Inf. Hiding Multimed. Signal Process.* **9**(2), 392–399 (2018).

[3] Thanki, R., Kothari, A. & Borra, S. Hybrid, blind and robust image watermarking: RDWT–NSCT based secure approach for telemedicine applications. *Multimed. Tools Appl.* **80**, 27593–27613. 10.1007/s11042-021-11064-y (2021).

[4] Alenizi, F. et al. Hybrid pyramid-DWT-SVD dual data hiding technique for videos ownership protection. *Multimed. Tools Appl.* **78**, 14511–14547. 10.1007/s11042-018-6723-9 (2019).

[5] Li, D. et al. A novel CNN based security guaranteed image watermarking generation scenario for smart city applications. *Inf. Sci.* **479**, 432–447. 10.1016/j.ins.2018.02.060 (2019).

[6] Wan, W. et al. Hybrid JND model-guided watermarking algorithm for screen content images. *Multimedia Tools Appl.* **79**, 4907–4930. 10.1007/s11042-018-6860-1 (2020).

[7] Prabha, K. & Sam, I. S. An effective robust and imperceptible blind color image watermarking using WHT. *J. King Saud Univ. Comput. Inf. Sci.* **34**(6), 2982–2992. 10.1016/j.jksuci.2020.04.003 (2022).

[8] Zear, A. & Singh, K. Secure and robust color image dual watermarking based on LWT-DCT-SVD. *Multimed. Tools Appl.* **81**, 26721–26738. 10.1007/s11042-020-10472-w (2022).

[9] Fan, X., Song, L. & Bao, S. Research on the application of digital watermarking technology in video data traceability. *Journal of Big Data and Computing*10.62517/jbdc.202301108 (2023).

[10] Mohanty, S., Sengupta,Guturu, A. & Kougianos, E. Everything You Want to Know About Watermarking: From Paper Marks to Hardware Protection, IEEE Consumer Electronics Magazine, 6,3, 83–91, (2017). 10.1109/MCE.2017.2684980

[11] Agarwal, N., Singh, A. K. & Singh, K. Survey of robust and imperceptible watermarking. *Multimed. Tools Appl.* **78**, 8603–8633. 10.1007/s11042-018-7128-5 (2019).

[12] Rhayma, H., Makhloufi, A., Hamam, H. & Hamida, A. B. Semi-fragile watermarking scheme based on perceptual hash function (PHF) for image tampering detection. *Multimedia Tools Appl.* **80**, 26813–26832. 10.1007/s11042-021-10886-0 (2021).

[13] Nguyen, T.-S. Fragile watermarking for image authentication based on DWT-SVD-DCT techniques. *Multim. Tools Appl.* **80**, 25107–25119. 10.1007/s11042-021-10879-z (2021).

[14] Roy, A. et al. An HVS inspired robust non-blind watermarking scheme in YCbCr color space. *Int. J. Image Graph.* **18**(3), 1850015. 10.1142/S0219467818500158 (2018).

[15] Liu, X., Lin, C. & Yuan, S. Blind dual watermarking for color images’ authentication and copyright protection. *IEEE Trans. Circuits Syst. Video Technol.* **28**(5), 1047–1055. 10.1109/TCSVT.2016.2633878 (2018).

[16] Palani, A. & Loganathan, A. Semi-blind watermarking using convolutional attention-based turtle shell matrix for tamper detection and recovery of medical images. *Expert Syst. Appl.* **238**, 121903. 10.1016/j.eswa.2023.121903 (2024).

[17] Abraham, J. & Paul, V. An imperceptible spatial domain color image watermarking scheme. *J. King Saud Univ. - Comput. Inf. Sci.* **31**(1), 125–133. 10.1016/j.jksuci.2016.12.004 (2019).

[18] Cedillo-Hernandez, M., Cedillo-Hernandez, A. & Garcia-Ugalde, F. J. Improving DFT-based image watermarking using particle swarm optimization algorithm. *Mathematics* **9**(15), 1795. 10.3390/math9151795 (2021).

[19] Yuan, Z. et al. New image blind watermarking algorithm based on two-dimensional discrete cosine transform. *Optik* **204** (164152). 10.1016/j.ijleo.2019.164152 (2020).

[20] Abdulrahman, A. K. & Ozturk, S. A novel hybrid DCT and DWT based robust watermarking algorithm for color images. *Multimedia Tools Appl.* **78**, 17027–17049. 10.1007/s11042-018-7085-z (2019).

[21] Hu, H. T., Hsu, L. Y. & Chou, H. H. An improved SVD-based blind color image watermarking algorithm with mixed modulation incorporated. *Inf. Sci.* **519**, 161–182. 10.1016/j.ins.2020.01.019 (2020).

[22] Su, Q. T. & Chen, B. J. A novel blind color image watermarking using upper Hessenberg matrix. *AEU Int. J. Electron. Commun.* **78**, 64–71. 10.1016/j.aeue.2017.05.025 (2017).

[23] Su, Q. T., Zhang, X. F. & Wang, G. An improved watermarking algorithm for color image using Schur decomposition. *Soft Comput.* **24**, 445–460. 10.1007/s00500-019-03924-5 (2020).

[24] Su, Q. T., Liu, Y. H., Liu, D. C., Yuan, Z. H. & Ning, H. Y. A new watermarking scheme for colour image using QR decomposition and ternary coding. *Multimed. Tools Appl.* **78**, 8113–8132. 10.1007/s11042-018-6632-y (2019).

[25] Ernawan, F. & Kabir, M. N. An improved watermarking technique for copyright protection based on Tchebichef moments. *IEEE Access* **7**, 151985–152003. 10.1109/ACCESS.2019.2948086 (2019).

[26] Venkataramana andA, A. & Raj Image watermarking using Krawtchouk moments, International Conference on Computing: Theory and Applications (ICCTA’07), IEEE, 676 – 68, (2007). 10.1109/ICCTA.2007.72

[27] Chekira, C., Marzouq, M., El Fadili, H., Lakhliai, Z. & da Graça Ruano, M. Join security and block watermarking-based evolutionary algorithm and Racah moments for medical imaging. *Biomed. Signal Process. Control* **96**, 106554. 10.1016/j.bspc.2024.106554 (2024).

[28] Sayyouri, M., Hmimid, A. & Qjidaa, H. Improving the performance of image classification by Hahn moment invariants. *J. Opt. Soc. Am. A* **30**(11), 2381–2394. 10.1364/JOSAA.30.002381 (2013). 10.1364/JOSAA.30.002381 24322939

[29] Li, L., Li, S., Abraham, A. & Pan, J. S. Geometrically invariant image watermarking using polar harmonic transforms. *Inf. Sci.* **199**, 1–19. 10.1016/j.ins.2012.02.062 (2012).

[30] Wang, Z., Bovik, A. C., Sheikh, H. R. & Simoncelli, E. Image quality assessment: From error visibility to structural similarity. *IEEE Trans. Image Process.* **13**(4), 600–612. 10.1109/TIP.2003.819861 (2004). 15376593 10.1109/tip.2003.819861

[31] Zhang, H. Y. A new image scrambling algorithm. *Int. Conf. Mach. Learn. Cybernetics*. **1088-1092**10.1109/ICMLC.2008.4620566 (2008).

[32] Lei, B., Soon, I. Y. & Tan, E. L. Robust SVD-based audio watermarking scheme with differential evolution optimization. *IEEE Trans. Audio Speech Lang. Process.* **21**(11), 2368–2378. 10.1109/TASL.2013.2277929 (2013).

[33] Eldaoushy, A. F. et al. Efficient hybrid digital image watermarking. *J. Opt.* **52**, 2224–2238. 10.1007/s12596-023-01144-7 (2023).

[34] Su, Q., Niu, Y., Wang, G., Jia, S. & Yue, J. Color image blind watermarking scheme based on QR decomposition. *Signal Process.* **94**, 219–235. 10.1016/j.sigpro.2013.06.025 (2014).

[35] Liu, J. et al. An optimized image watermarking method based on HD and SVD in DWT domain. *IEEE Access* **7**, 80849–80860. 10.1109/ACCESS.2019.2915596 (2019).

[36] Fouda, Y. M. Watermarking and reconstructing multi-channel images in the transform domain with application to diabetic retinopathy medical images. *Jordan J. Electr. Eng.* **11**(4), 776–805 (2025).

[37] Singh, R., Pal, R. & Joshi, D. Optimal frame selection-based watermarking using a meta-heuristic algorithm for securing video content. *Comput. Electr. Eng.* **121**, 109857. 10.1016/j.compeleceng.2024.109857 (2025).

[38] Ujwala, N., Kumar, S., Ram, J. Y. S., Kumar, J. L. & Choudhary, K. H. R. Alpha Blending-Based Adaptive Color Image Watermarking Technique, in Proc. Int. Conf. Generative AI, Cryptography and Predictive Analytics (ICGCPA), Singapore: Springer Nature, 415–426, (2025). 10.1007/978-981-97-9132-3_27

[39] Singh, R., Pal, R., Mittal, H. & Joshi, D. Multi-objective optimization-based medical image watermarking scheme for securing patient records. *Comput. Electr. Eng.* **118**, 109303. 10.1016/j.compeleceng.2024.109303 (2024).

[40] Sharma, S., Sharma, H. & Sharma, J. B. An adaptive color image watermarking using RDWT-SVD and artificial bee colony based quality metric strength factor optimization. *Appl. Soft Comput.* **84**, 105696. 10.1016/j.asoc.2019.105696 (2019).

[41] Singh, R. et al. From classical to soft computing based watermarking techniques: A comprehensive review. *Future Gener. Comput. Syst.* **141**, 738–754. 10.1016/j.future.2022.12.015 (2023).

[42] Sharma, S., Sharma, H., Sharma, J. B. & Poonia, R. C. A secure and robust color image watermarking using nature-inspired intelligence. *Neural Comput. Appl.* **33**(7), 4919–4937. 10.1007/s00521-020-05634-8 (2023).

[43] Singh, R., Shukla, K., Kumar, T. & Kapse, V. M. Robust medical image watermarking in frequency domain. *Int. J. Electr. Electron. Res.* **11**(3), 859–865. 10.37391/ijeer.110333 (2023).

[44] Kumar, S. et al. Entropy based adaptive color image watermarking technique in YCbCr color space. *Multim. Tools Appl.* **83**, 13725–13751. 10.1007/s11042-023-16059-5 (2024).

[45] Sharma, S., Sharma, H. & Sharma, J. B. A new optimization based color image watermarking using non-negative matrix factorization in discrete cosine transform domain. *J. Ambient Intell. Human. Comput.* **12**, 4297–4319. 10.1007/s12652-021-03408-1 (2022).

[46] Singh, R., Ashok, A. & Saraswat, M. High embedding capacity based color image watermarking scheme using SBBO in RDWT domain. *Tools Appl.* **82**, 3397–3432. 10.1007/s11042-022-13286-0 (2023).

[47] https://www.imageprocessingplace.com/root_files_V3/image_databases.htm

[48] http://sipi.usc.edu/database/

[49] https://www.kaggle.com/datasets/shuvokumarbasak2030/brain-tumor-mri-colorized-dataset

[50] https://www.kaggle.com/datasets/shuvokumarbasak2030/medical-imaging-ct-xray-colorization-new-dataset

[51] https://www.kaggle.com/datasets/nguyenhung1903/diaretdb1-standard-diabetic-retinopathy-database

[52] https://www.kaggle.com/datasets/programmer3/aerial-landscapes-dataset/data

